# Fibroblast-derived extracellular vesicles contain SFRP1 and mediate pulmonary fibrosis

**DOI:** 10.1172/jci.insight.168889

**Published:** 2024-08-15

**Authors:** Olivier Burgy, Christoph H. Mayr, Déborah Schenesse, Efthymios Fousekis Papakonstantinou, Beatriz Ballester, Arunima Sengupta, Yixin She, Qianjiang Hu, Maria Camila Melo-Narvaéz, Eshita Jain, Jeanine C. Pestoni, Molly Mozurak, Adriana Estrada-Bernal, Ugochi Onwuka, Christina Coughlan, Tanyalak Parimon, Peter Chen, Thomas Heimerl, Gert Bange, Bernd T. Schmeck, Michael Lindner, Anne Hilgendorff, Clemens Ruppert, Andreas Güenther, Matthias Mann, Ali Önder Yildirim, Oliver Eickelberg, Anna Lena Jung, Herbert B. Schiller, Mareike Lehmann, Gerald Burgstaller, Melanie Königshoff

**Affiliations:** 1 INSERM U1231 Center for Translational and Molecular Medicine (CTM), Faculty of Health Sciences, Université de Bourgogne, Dijon, France.; 2 Reference Center for Rare Pulmonary Diseases, University Hospital Dijon-Bourgogne, Dijon, France.; 3 Institute of Experimental Pneumology, LMU University Hospital, Ludwig-Maximilians University, Munich, Germany.; 4 Department of Pulmonary Medicine and Intensive Care Unit, University Hospital Dijon-Bourgogne, Dijon, France.; 5 Institute for Lung Research, Philipps-University Marburg, German Center for Lung Research (DZL), Marburg, Germany.; 6 Comprehensive Pneumology Center (CPC) with the CPC-M BioArchive and Institute of Lung Health and Immunity (LHI), Helmholtz Munich, Member of the DZL, Munich, Germany.; 7 Faculty of Health Sciences, Universidad CEU Cardenal Herrera, CEU Universities, Valencia, Spain.; 8 Center for Lung Aging and Regeneration (CLAR), Division of Pulmonary, Allergy, Critical Care and Sleep Medicine, Department of Medicine, University of Pittsburgh, Pittsburgh, Pennsylvania, USA.; 9 Division of Neurology, Department of Medicine, University of Colorado Denver, Aurora, Colorado, USA.; 10 Women’s Guild Lung Institute, Division of Pulmonary and Critical Care Medicine, Department of Medicine, Cedars-Sinai Medical Center, Los Angeles, California, USA.; 11 Center for Synthetic Microbiology (SYNMIKRO) and; 12 Core Facility Flow Cytometry – Bacterial Vesicles, Philipps-University Marburg, Marburg, Germany.; 13 Universities of Giessen and Marburg Lung Center (UGMLC) Giessen Biobank, Justus-Liebig-University Giessen, DZL, Giessen, Germany.; 14 Paracelsus Medical Private University, Salzburg, Austria.; 15 European IPF Registry (eurIPFreg), Center for Interstitial and Rare Lung Diseases, UGMLC, Justus-Liebig University Giessen, DZL, Giessen, Germany.; 16 Department of Proteomics and Signal Transduction, Max Planck Institute of Biochemistry, Martinsried, Germany.; 17 Research Unit for Precision Regenerative Medicine, Helmholtz Munich, Munich, Germany.; 18 Geriatric Research Education and Clinical Center (GRECC) at the VA Pittsburgh Healthcare System, Pittsburgh, Pennsylvania, USA.

**Keywords:** Cell biology, Pulmonology, Fibrosis, Molecular biology

## Abstract

Idiopathic pulmonary fibrosis (IPF) is a lethal chronic lung disease characterized by aberrant intercellular communication, extracellular matrix deposition, and destruction of functional lung tissue. While extracellular vesicles (EVs) accumulate in the IPF lung, their cargo and biological effects remain unclear. We interrogated the proteome of EV and non-EV fractions during pulmonary fibrosis and characterized their contribution to fibrosis. EVs accumulated 14 days after bleomycin challenge, correlating with decreased lung function and initiated fibrogenesis in healthy precision-cut lung slices. Label-free proteomics of bronchoalveolar lavage fluid EVs (BALF-EVs) collected from mice challenged with bleomycin or control identified 107 proteins enriched in fibrotic vesicles. Multiomic analysis revealed fibroblasts as a major cellular source of BALF-EV cargo, which was enriched in secreted frizzled related protein 1 (SFRP1). *Sfrp1* deficiency inhibited the activity of fibroblast-derived EVs to potentiate lung fibrosis in vivo. SFRP1 led to increased transitional cell markers, such as keratin 8, and WNT/β-catenin signaling in primary alveolar type 2 cells. SFRP1 was expressed within the IPF lung and localized at the surface of EVs from patient-derived fibroblasts and BALF. Our work reveals altered EV protein cargo in fibrotic EVs promoting fibrogenesis and identifies fibroblast-derived vesicular SFRP1 as a fibrotic mediator and potential therapeutic target for IPF.

## Abstract 2

Fibroblasts secrete altered extracellular vesicles loaded with SFRP1 that drive lung fibrosis

## Graphical abstract

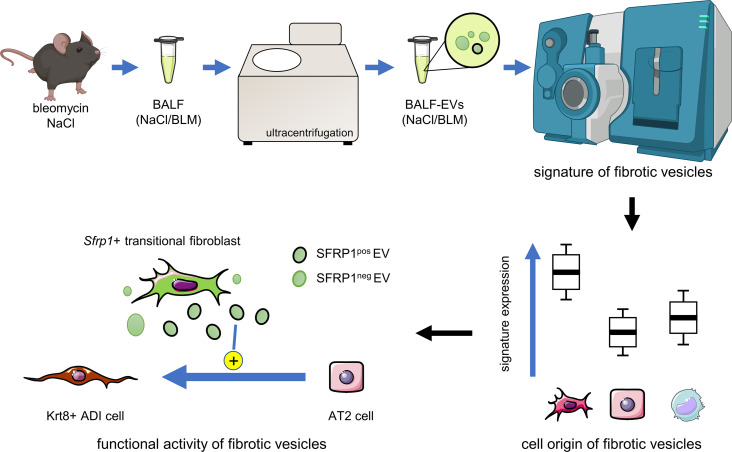

## Introduction

Fibroproliferative diseases affect all tissues and organ systems. They represent a major health problem and are responsible for 45% of deaths around the world (1). Among them, idiopathic pulmonary fibrosis (IPF) is a chronic progressive and fatal fibrotic disorder of the lung (2). Current therapies are limited to 2 approved drugs, pirfenidone and nintedanib, which slow down the progression of disease but are unable to stop or reverse it (3, 4). Thus, there is a major unmet clinical need for targeted therapies. IPF is thought to be driven by repetitive insults to the lung epithelium that result in a local pro-fibrotic milieu within the lung where fibroblasts, the key effector cells in fibrosis, are activated and lead to increased extracellular matrix deposition (2). The (re)activation of developmental signaling pathways, such as TGF-β or WNT, leads to impaired cell-to-cell communication resulting in tissue fibrosis and scarring (5–7). The mechanisms involved in cellular crosstalk contributing to fibrosis, however, are still poorly understood. In addition, whether their inhibition can mitigate fibrosis development and progression remains largely unknown.

Extracellular vesicles (EVs) have emerged as potent contributors to cellular crosstalk in several diseases (8). They represent a group of membranous structures with a size range from 30 to 1,000 nm depending on origin and are secreted by all cell types (9). EVs contain a wide array of cargo, from proteins to nucleic acids and lipids, and thus are major players in cellular crosstalk (10). The diverse cargo transported by the vesicles mediates the biological activity of EVs; however, their composition and distinct effects in fibrosis in general, and in pulmonary fibrosis in particular, are poorly understood (5, 11–16). We and others have recently demonstrated that EVs are increased in several bodily fluids, such as bronchoalveolar lavage fluid (BALF), sputum, plasma, or urine, in experimental lung fibrosis as well as in human IPF (17, 18). During fibrosis, EVs carry specific nucleic acids such as miRNAs, which can alter pro-fibrotic signaling in IPF (17–20). Moreover, we found that EVs carry WNT5A, which potentiates profibrotic fibroblast function (17). Together, these data strongly support the notion that EVs carry distinct cargo as impactful mediators of fibrosis, which might be amenable for therapeutic targeting.

To gain a deeper understanding of the altered EV cargo in pulmonary fibrosis, and their potential as novel therapeutic targets and biomarkers, we performed an unbiased analysis of the EV proteome in lung fibrosis. We found a distinct profile of proteins enriched specifically in fibrotic EVs and showed that fibrotic EVs impair lung epithelial stem cell function and modulate extracellular matrix deposition. We discovered that fibroblasts are key cells secreting EVs during murine and human lung fibrosis. These cells secrete EVs loaded with secreted frizzled related protein 1 (SFRP1), a WNT family member protein, enriched in fibrotic EVs. We show that EV-bound SFRP1 derived from fibroblasts contributes to impaired alveolar epithelial cell differentiation and exaggerates lung fibrosis in vivo. SFRP1 deletion in fibroblasts was sufficient to inhibit the pro-fibrotic properties of their vesicles in vivo, thus uncovering a potential therapeutic EV-linked target protein in pulmonary fibrosis.

## Results

### EVs accumulate during active fibrosis and drive pro-fibrotic mechanisms.

**Figure 1:**
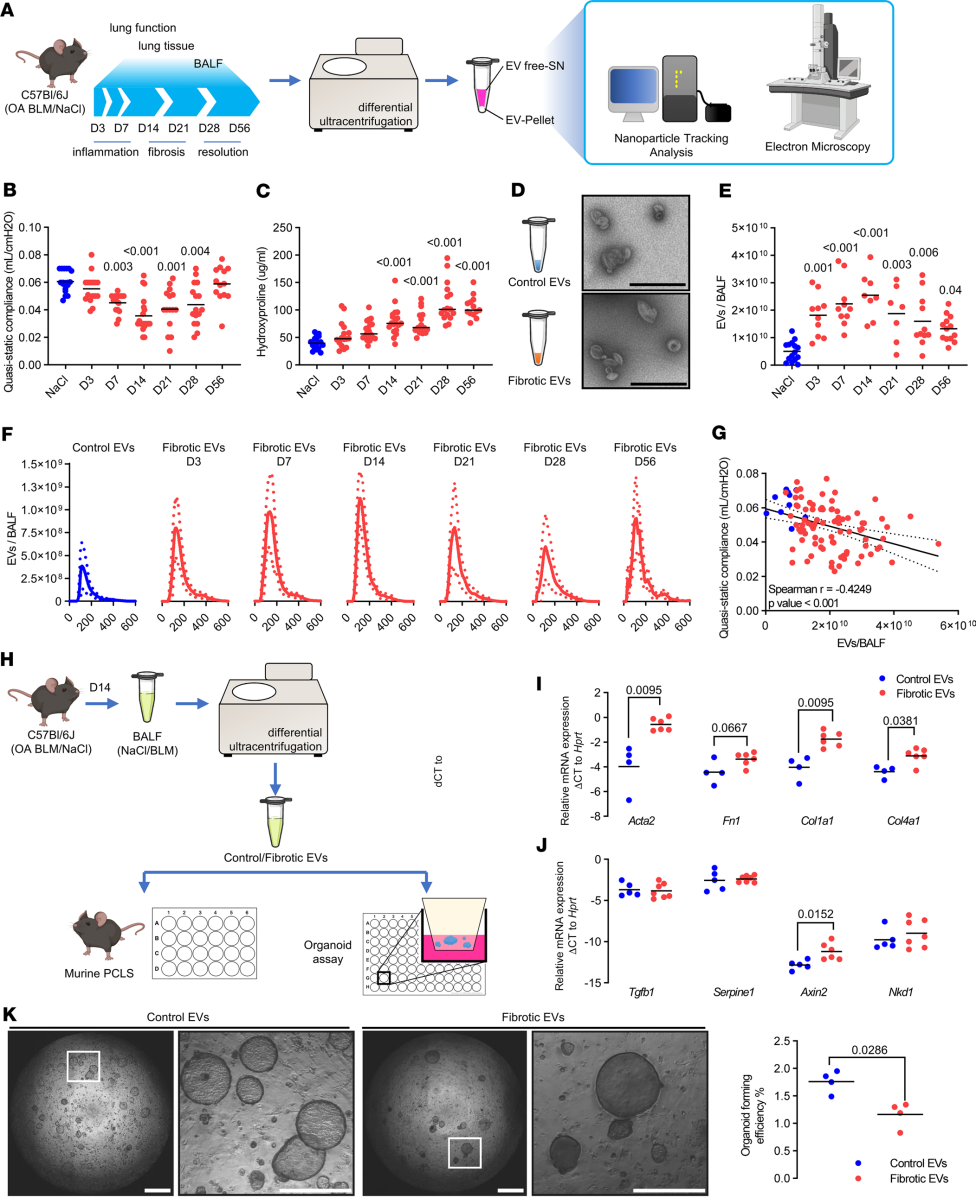
EVs accumulate in lung fibrosis, initiate lung remodeling, and impair alveolar epithelial cell function. (**A**) C57BL/6J mice were exposed to orotracheal bleomycin or NaCl (control). Lung function was assessed and lung tissue and BALF were collected over the indicated time course. EVs were concentrated from BALF and characterized. BLM, bleomycin. (**B**) Quasistatic compliance and (**C**) hydroxyproline level of the corresponding experiments are shown. (**D**) BALF-EVs were observed by electron microscopy (scale bars indicate 600 nm) and (**E**) numbered by Nanosight (data expressed as number of particles per BALF). (**F**) EV quantification according to particle diameters (expressed in nm) at each time point after bleomycin exposure (mean ± SD). (**G**) Correlation between EV number and quasistatic compliance is depicted. (**B**–**G**) Each point corresponds to a mouse (*n* = 5–8 for NaCl groups, *n* = 13–20 for BLM groups). (**H**) BALF-EVs were isolated from mice with pulmonary fibrosis (14 days after bleomycin) or control mice and used for functional assays. (**I** and **J**) PCLS from normal C57BL/6J were cultured with the abovementioned BALF-EVs. After 7 days, the expression of fibrosis-related genes was assessed by qPCR. Data are representative of PCLS from individual mice exposed to control- (*n* = 4 PCLS) or fibrotic EVs (*n* = 6 PCLS). Gene expression was normalized to *Hprt* expression. (**K**) Murine EpCAM-positive cells and CCL-206 fibroblasts in Matrigel were exposed to BALF-EVs for 14 days. Representative images (left panel, scale bar = 1 mm or 500 μm for region of interest [ROI] zoom) and quantification (right panel, *n* = 4 control EVs, *n* = 4 fibrotic EVs) of the organoid formation efficiency. Statistical analysis by Kruskal-Wallis followed by Dunn’s multiple comparisons tests (**B**–**D**), Spearman’s correlation test (**G**), or nonparametric Mann-Whitney test (**I**–**K**). *P* values are indicated for each comparison.

We first assessed the quantity of EVs secreted during lung fibrosis initiation, progression, and resolution using a well-known experimental mouse model of lung fibrosis, in which C57BL/6J mice are challenged with a single orotracheal instillation of bleomycin (day 0) (Figure 1A). EVs were characterized over an in-depth time course including the phase of inflammation (days 3–7, D3–D7), followed by fibrosis (days 10–21) and late resolution of the fibrotic lesions (days 28–56) (Figure 1A) (21, 22). Pulmonary fibrosis was assessed by lung function and hydroxyproline content of the lung tissue, demonstrating active fibrosis at day 14 (Figure 1, B and C), consistent with previous reports (23). BALF was collected at different time points after bleomycin (from D3 to D56), and BALF-derived EVs (BALF-EVs) were concentrated by differential ultracentrifugation using a previously validated protocol (17, 19, 24). Electron microscopy validated the presence of EVs with a size consistent with small EVs and the typical cup-shaped morphology observed under transmission electron microscopy (TEM) (Figure 1D). EVs were consistently increased in BALF samples at all time points after bleomycin challenge compared with saline-treated controls, with a peak at day 14, as quantified by nanoparticle tracking analysis (NTA) (Figure 1E). Consistent with previous reports, sampled EVs had a median size ranging from 103 to 190 nm in diameter (95% confidence interval) (Figure 1F) (17, 19). Notably, the quantity of EVs present at the BALF level correlated inversely with the lung function of mice exposed to bleomycin, with the most severe lung function decline at day 14 (Figure 1G).

We aimed to establish the functional relevance of EVs for fibrogenesis in the lung. To this end, EVs were concentrated from the BALF of mice exposed to bleomycin for 14 days (called fibrotic EVs from here on) or saline control (which we will refer to as control EVs). The functional properties of these vesicles were tested by assessing profibrotic markers in multicellular precision-cut lung slices (PCLS) and alveolar epithelial type 2 (AT2) stem cell–based organoid assays (Figure 1H). PCLS generated from naive C57BL/6J mice were treated with fibrotic or control EVs, respectively, and fibrotic markers were assessed by quantitative PCR (qPCR). Notably, fibrotic but not control EVs induced the expression of extracellular matrix and fibrosis markers such as α–smooth muscle actin (α-SMA) (*Acta2*, *P* value 0.0095), fibronectin (*Fn1*, *P* value 0.0667), type 1 collagen (*Col1a1*, *P* value 0.0095), and type 4 collagen (*Col4a1*, *P* value 0.0381) in healthy PCLS ex vivo (Figure 1I). Moreover, WNT signaling is known to be aberrantly active in pulmonary fibrosis (6), and we found that the WNT target gene *Axin2* was significantly upregulated by fibrotic EVs compared to control EVs (Figure 1J). Impaired lung epithelial cell function is another hallmark of pulmonary fibrosis (25–27). The progenitor cell function of epithelial AT2 stem cells is reduced in pulmonary fibrosis (15, 28). In line with this, we found significantly fewer organoids when healthy murine AT2 cells were exposed to fibrotic EVs versus control vesicles (Figure 1K). These findings support the notion that fibrotic EVs carry cargo that is sufficient to initiate and/or drive fibrosis.

### Fibrotic EVs have a distinct proteomic profile.

**Figure 2:**
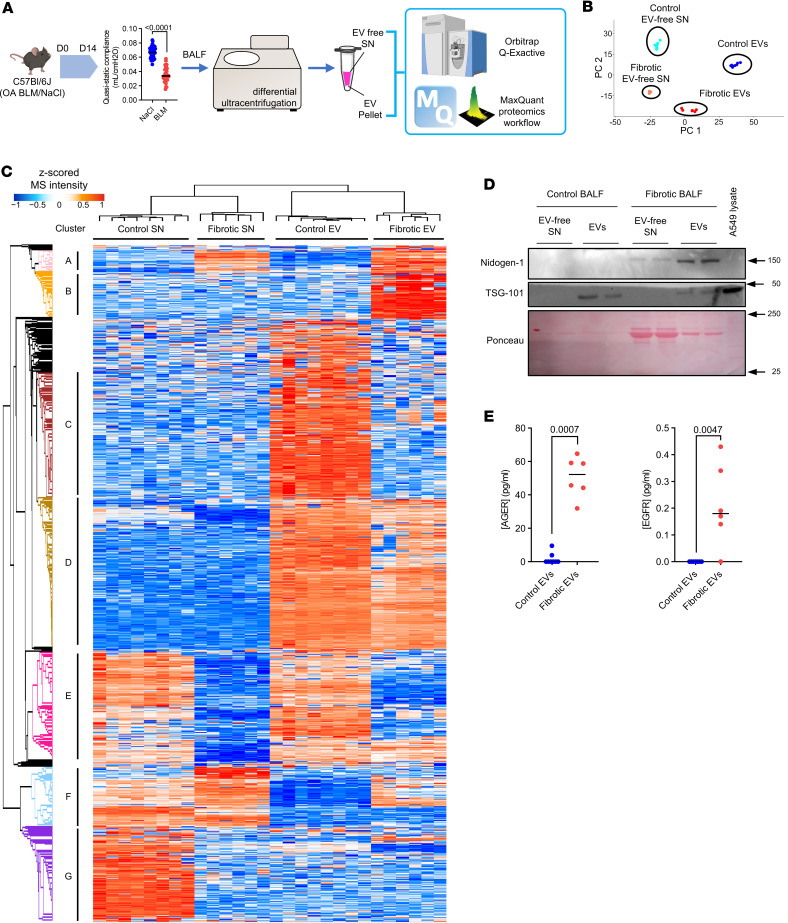
Label-free proteomics identifies several proteins specific to fibrotic EVs. (**A**) C57BL/6J mice were exposed to orotracheal bleomycin or NaCl as control. At 14 days after injection, lung function was assessed and BALF was collected. BALF was utilized for EV isolation, and vesicular (EV pellet) as well as nonvesicular counterparts (EV-free SN) were subjected to label-free proteomics (*n* = 8 for control, *n* = 6 for bleomycin). (**B**) Principal component analysis representation of the different samples. (**C**) Heatmap of the identified proteins. Color coding corresponds to *z*-scored MS intensity values after imputation. Based on the unsupervised clustering, proteins were grouped into 7 clusters called A to G. (**D**) Levels of nidogen-1 (Western blot) in EVs from bleomycin or control mice are shown. TSG-101 was used to show protein enrichment in EVs, and protein content for each sample is shown with Ponceau. A549 lysate served as positive control. Equal (10 μg) protein content was used for the Western blot. (**E**) AGER and EGFR levels assessed by ELISA on normal (blue) and fibrotic (red) EVs. Data are presented as analyte concentration (pg/mL) normalized to 2 × 10^8^ vesicles. Statistical analysis by nonparametric Mann-Whitney. SN, EV-free fraction.

To identify the cargo responsible for the pro-fibrotic activity of EVs, we comprehensively characterized their proteomic profile. We generated BALF-EVs and the corresponding EV-free BALF fractions, or EV-free supernatant (SN), from fibrotic and healthy mouse lungs, respectively (Figure 2A). After quality control by NTA (Supplemental Figure 1A; supplemental material available online with this article; https://doi.org/10.1172/jci.insight.168889DS1), the samples were subjected to unbiased label-free shotgun proteomics. This approach identified 1,634 proteins overall, including 774 proteins specifically enriched in the EV samples (94 identified only in fibrotic EVs, 389 identified only in control EVs), and 218 specific to the EV-free fractions (Supplemental Figure 1B). Both principal component analysis and the heatmap generated after unsupervised hierarchical clustering of the samples based on Pearson’s correlation revealed significant differences in the proteomic profiles among all samples (Figure 2, B and C). Importantly, we observed enrichment for proteins classically identified in EVs (as described in Vesiclepedia, http://www.microvesicles.org) in the vesicular fractions compared with EV-free fractions, including CD9, apoptosis-linked gene (ALG)-2 interacting protein X (ALIX), flotillin-1, or TSG101, validating our EV isolation protocol (Supplemental Figure 1C). Moreover, fibrotic EVs showed an enrichment in several proteins involved in fibrogenesis markers, including tenascin C, MMP19, and collagens, compared with control EVs (Supplemental Figure 1D). By unsupervised clustering, we identified 7 specific clusters demonstrating distinct protein profiles between control and fibrosis, and remarkably, also between EVs and EV-free samples (Figure 2C). These clusters were enriched for specific biological processes based on Gene Ontology (GO) terms (Supplemental Figure 2A). Cluster D illustrated the differences between EVs and EV-free fractions and was composed mainly of proteins important for EV generation and secretion, such as Rab proteins, caveolin, and flotillins (Supplemental Figure 2A). Cluster A showed extracellular proteins, both EV-linked or free, secreted upon bleomycin exposure compared with NaCl controls (Figure 2C). This cluster was enriched in proteins belonging to TGF-β signaling (Tgfbi) and extracellular matrix (TnC) (Supplemental Figure 2A). More importantly, cluster B contained 107 EV proteins, which were specifically enriched in fibrotic EVs compared with normal EVs and were not changed in the corresponding EV-free SN fraction (Figure 2C). This cluster B highlighted proteins linked to GO terms such as cell adhesion, extracellular matrix organization, developmental processes, or cell communication and proliferation, and we observed significant enrichment for fibrosis-relevant GO terms such as extracellular matrix organization (FDR 3.13 × 10^–7^), cell-substrate adhesion (FDR 1.96 × 10^–9^), and wound healing (FDR 2.28 × 10^–6^) (Table 1, Table 2, and Supplemental Figure 2, B and C). Analysis of the reactome of these fibrotic EV proteins highlighted pathways such as laminin interactions, MET signaling, cell motility, and degradation of the extracellular matrix (Supplemental Figure 2D). Among the proteins identified in cluster B, nidogen-1, advanced glycosylation end-product specific receptor (AGER), and epidermal growth factor receptor (EGFR) were previously linked to the mechanisms of fibrogenesis (29, 30) and found to be secreted by cells via EVs (31–33). We validated the specific enrichment of these 3 proteins identified by our unbiased proteomic approach during lung fibrosis and in fibrotic EVs (Figure 2, D and E, and Supplemental Figure 3).

### Fibroblasts are a major source of EVs containing SFRP1 in lung fibrosis.

**Figure 3:**
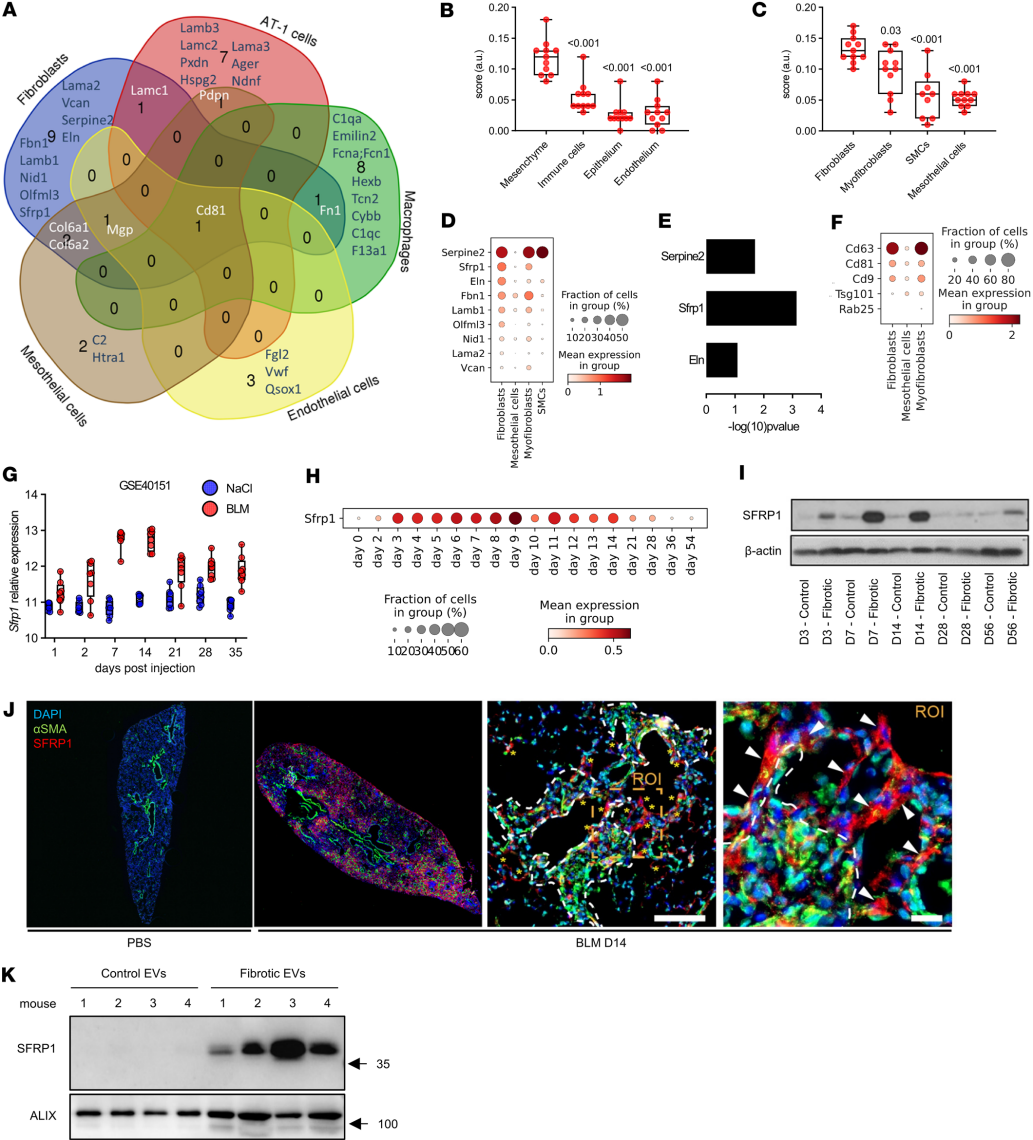
Fibroblasts are a major source of EVs during fibrosis. (**A**) Venn diagram depicting the cellular origin of the bleomycin BALF-EV proteins. (**B** and **C**) Scoring of an scRNA-Seq dataset (GSE141259) for the mean expression of the 107 proteins identified in the bleomycin BALF-EVs in the main cellular compartments of the lung (**B**) as well as in mesenchymal populations (**C**). Box plots show the interquartile range, median (line), and minimum and maximum (whiskers). SMCs, smooth muscle cells. (**D**) Top proteins expressed in fibroblasts, among the proteins identified in bleomycin-BALF-EV and classified in main mesenchymal cellular compartments. (**E**) Statistical difference in the proteomic dataset between fibrotic and control EVs for the top 3 most expressed fibroblast-related genes. (**F**) Analysis of an scRNA-Seq dataset (GSE40151) for the expression of EV machinery in fibroblasts expressing (true) or not expressing (false) *Sfrp1*. (**G**) Gene expression of *Sfrp1* in the lungs of mice with bleomycin-induced lung fibrosis or control (NaCl) mice. Data from GSE40151. (**H** and **I**) Expression of SFRP1 in lung tissue from mice exposed to bleomycin at different time points at the transcriptomic (**H**, data from GSE141259) or proteomic level (**I**). (**J**) Immunofluorescence staining for α-SMA (green) and SFRP1 (red) in FFPE lung sections from mice challenged with bleomycin (day 14) or NaCl as control. Representative observation of a fibrotic area from a mouse lung 14 days after bleomycin (right panels). Asterisks denote SFRP1^+^ transitional fibroblasts, dashed lines indicate fibrotic dense areas with α-SMA^+^ myofibroblasts (in green), and arrowheads point out single SFRP1^+^ transitional fibroblasts (in red) in the zoomed ROI. Nuclei are stained with DAPI (blue). Scale bars = 100 μm or 20 μm (ROI’s zoom). (**K**) Western blot for SFRP1 expression on normal and fibrotic EVs (*n* = 4/group). Equal number of vesicles (2 × 10^8^) loaded. ALIX serves as EV-enriched protein. Molecular weights (kDa) are indicated. All statistical analyses by nonparametric Mann-Whitney. *P* values as indicated.

IPF results from an impaired cellular crosstalk involving aberrantly activated epithelial, mesenchymal, and immune cells, respectively (2). We thus aimed to shed light on the key cellular origin(s) of the EVs by annotating the proteins from our proteomic dataset using a single-cell RNA-Seq (scRNA-Seq) dataset of the bleomycin model, which we recently published (34). Cluster B contained 107 proteins significantly enriched in fibrotic EVs compared with control vesicles. From these proteins, 47 proteins were linked to a specific cellular annotation, including alveolar epithelial cells, macrophages, or fibroblasts, in this existing scRNA-Seq dataset. Notably, we found that the highest number of proteins had a known annotation for fibroblasts (Figure 3A). This suggests that fibroblasts are a major, though likely not the only, producers of vesicles found in fibrotic BALF. To further corroborate this, we used the same scRNA-Seq dataset to investigate which cell type expressed the highest level of the whole fibrotic EV protein signature (107 proteins of cluster B). We scored each cell type according to the expression of protein signatures identified in control EVs, fibrotic EVs, or in both EV groups together (Figure 3B and Supplemental Figure 4). Consequently, our analysis revealed that mesenchymal cells are the main source of a 107-protein–containing signature specifically enriched in fibrotic EVs (Figure 3B). Of note, similar differences were not observed for the expression of control EVs or EV-specific proteins, for which the overall expression remained stable across the cell clusters (Supplemental Figure 4, C and D). Moreover, within these stromal cell types, fibroblasts were identified as the main source expressing the fibrotic EV protein signature (Figure 3C). We thus focused on fibroblasts and ranked the fibroblast-specific EV proteins according to their expression levels. This analysis revealed that from the originally identified fibrotic EV protein signature, *Serpine2*, *Sfrp1*, and *Eln* were the 3 highest ranked genes expressed in fibroblasts (Figure 3D). Among fibroblast subpopulations, *Sfrp1* was recently shown to be distinctly expressed in transitional fibroblasts, which precede ACTA2/SPP1/CTHRC1^+^ myofibroblasts and thus contribute to lung repair and fibrosis (Supplemental Figure 5) (35–37). Within our proteomic data set, SFRP1 was the most significantly enriched protein in fibrotic EVs compared with control vesicles (Figure 3E). In addition, *Sfrp1*-expressing fibroblasts had enhanced expression of the machinery for EV/exosome biogenesis (Figure 3F), suggesting they may produce more vesicles. We therefore focused our analysis on SFRP1, a known regulator of WNT signaling (6), which is altered in fibrosis (36, 38). Microarray analysis of a bleomycin time course experiment (National Center for Biotechnology [NCBI] Gene Expression Omnibus [GEO] accession GSE40151) showed that *Sfrp1* transcript expression increased in mouse lungs starting 7 days after bleomycin until day 14 compared with corresponding saline controls before decreasing after day 21 (Figure 3G). We verified this kinetic using an scRNA-Seq dataset our group published (34), where *Sfrp1* increased as early as day 3 after bleomycin challenge, with a peak of expression between day 9 and day 11, before returning to basal level after 21 days (Figure 3H). We verified the transcriptomic data by Western blotting demonstrating elevated Sfrp1 in whole lungs day 7 to day 14 after bleomycin instillation compared with saline control (Figure 3I). On immunofluorescence-stained lung sections, Sfrp1 localized to regions of active fibrosis containing α-SMA–positive myofibroblasts (Figure 3J), which is in line with our previous findings of Sfrp1^+^ transient fibroblasts (36). Interestingly, within the 5 members of the SFRP family, *SFRP1* was the highest expressed isoform in lung fibroblasts (Supplemental Figure 6). Finally, we also verified increased SFRP1 protein expression in fibrotic BALF-EVs isolated 14 days after bleomycin challenge compared with control vesicles (Figure 3K).

### SFRP1 promotes the pro-fibrotic activities of EVs in vivo.

**Figure 4:**
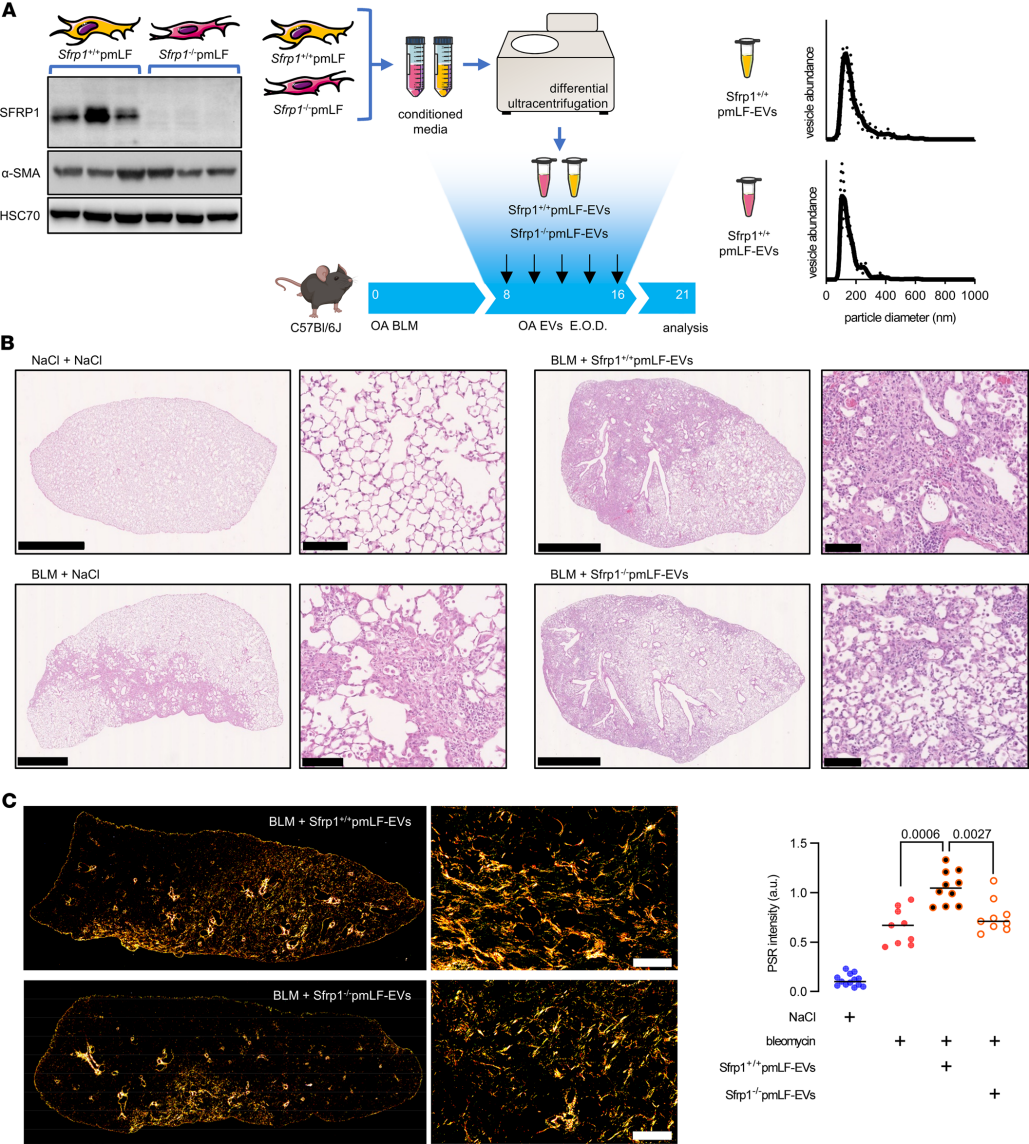
*Sfrp1* deficiency in fibroblast-derived EVs attenuates lung fibrosis in vivo. (**A**) General outline of the experiment. EVs were isolated from the conditioned media of primary mouse lung fibroblasts (pmLFs) isolated from mice with genetic deletion of Sfrp1 (Sfrp1^–/–^) or WT control (Sfrp1^+/+^). SFRP1 expression was verified by Western blot (left panel). Isolated vesicles were characterized for size by NTA and injected intratracheally in mice previously challenged with bleomycin or control NaCl. E.O.D., every other day. (**B**) Representative histology of the lung of the above-described mice at D21 after bleomycin exposure. Scale bars indicate 2.5 μm or 100 μm (zoom). (**C**) Collagen quantification on FFPE lung sections stained with Picrosirius red and visualized under polarized light. Representative observation (*n* = 10 for BLM+Sfrp1^+/+^ pmLF-EVs and *n* = 9 BLM+Sfrp1^–/–^ pmLF-EVs, left panel. Scale bar = 100 μm) and quantification (right panel) are shown. Statistical analysis by nonparametric Mann-Whitney. Each point represents 1 mouse. *P* values are indicated for each comparison.

We next aimed to determine the functional effect of SFRP1 in fibroblast-EVs on already established lung fibrosis in vivo. To this end, we used primary adult lung fibroblasts isolated from *Sfrp1*-deficient (*Sfrp1*^–/–^) mice or control (*Sfrp1*^+/+^) mice (Figure 4A). EVs were concentrated from the conditioned media of *Sfrp1*^–/–^ and *Sfrp1*^+/+^ fibroblasts and instilled intratracheally into mice previously challenged with bleomycin, based on a previously published protocol (19). EVs were administered repeatedly starting day 8 after bleomycin as outlined in Figure 4A. At day 21, we observed that EVs from control fibroblasts significantly exaggerated tissue remodeling within the distal lung, while the mice receiving intratracheal EVs from *Sfrp1^–/–^* fibroblasts exhibited substantially less tissue fibrosis compared with the EVs derived from *Sfrp1*^+/+^ fibroblasts (Figure 4B). Accordingly, mice exposed to EVs from *Sfrp1*^–/–^ fibroblasts had significantly less lung collagen accumulation compared with mice receiving EVs from *Sfrp1*^+/+^ control fibroblasts as assessed by Picrosirius red staining (Figure 4C).

### SFRP1-EVs promote the accumulation of keratin 8–positive expressing AT2 cells.

**Figure 5:**
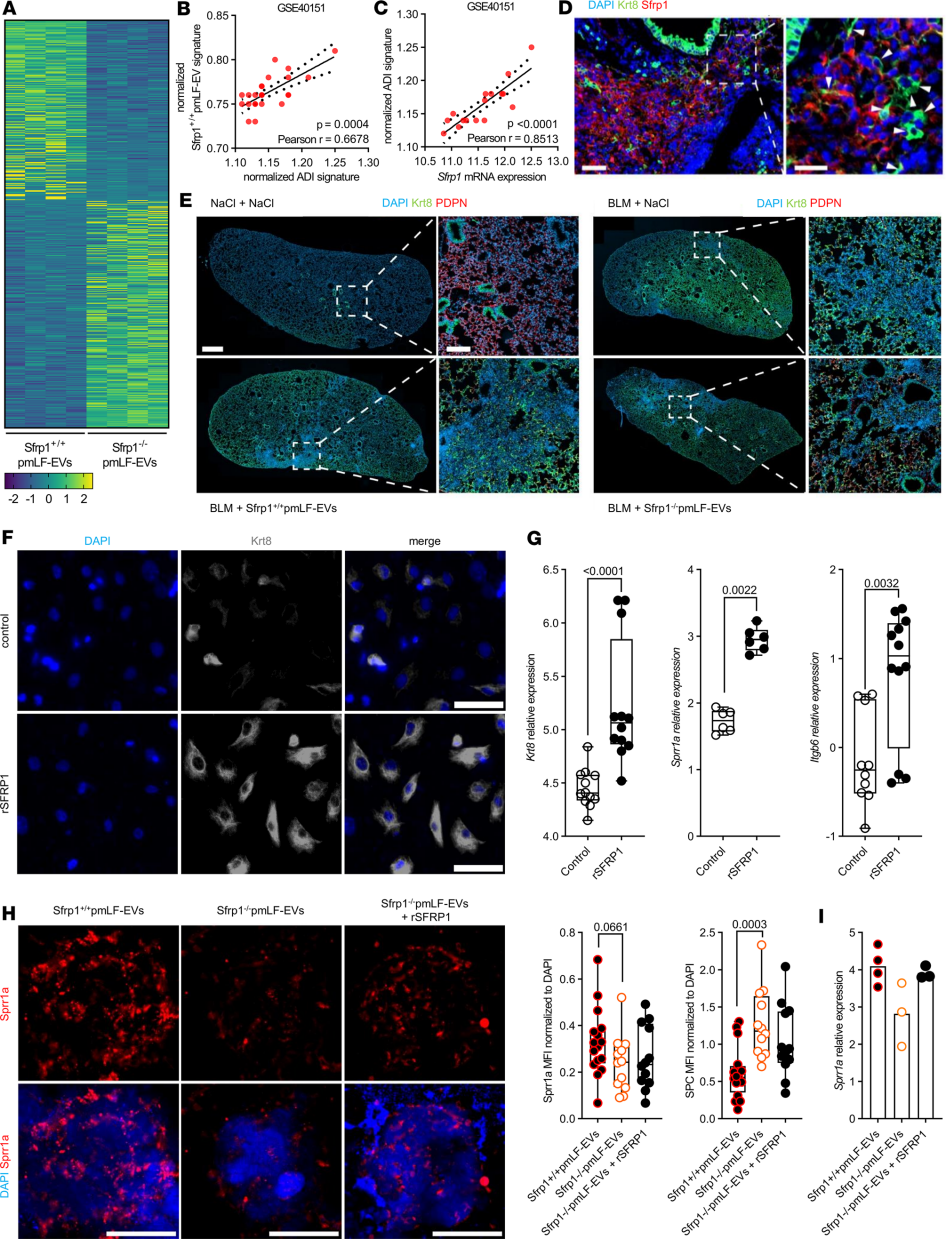
SFRP1-EVs promote the accumulation of keratin 8–positive expressing AT2 cells. (**A**) Heatmap of differentially expressed genes (*P* < 0.05) between WT EV and KO EV groups (*n* = 4 samples/group). Genes are ranked by averaged decreasing expression in the WT EV group. Krt8, keratin 8. (**B** and **C**) Correlation of WT EV signature (top 10) versus the ADI gene signature (**B**) or Sfrp1 expression versus ADI gene signature (**C**). Data from GSE40151 (D14 to D28 after bleomycin, *n* = 24). (**D**) Immunofluorescence analysis of lung tissue sections from bleomycin-treated mice at day 14 after injury (BLM D14) displaying the appearance of SFRP1^+^ cells (red) surrounding Krt8^+^ expressing ADI cells (green). Arrowheads in the magnified inset point out single Krt8^+^ ADI cells. Nuclei stained with DAPI (blue). Scale bars = 20 μm. (**E**) Immunofluorescence analysis of lung tissue sections from bleomycin-treated mice at day 14 after injury (BLM) compared with healthy controls (NaCl). BLM-treated mice were additionally treated with/without SFRP1-containing EVs. Podoplanin (PDPN) in red, Krt8 in green, and nuclei stained with DAPI in blue. Scale bars = 1,000 μm and 200 μm (ROI). (**F** and **G**) pmAT2 cells were cultured with or without recombinant (r) SFRP1. After 6 days, cells were analyzed for the expression of Krt8, Sprr1a, or Itgb6 by immunofluorescence (**F**) or qPCR (**G**). Scale bars = 50 μm. Representative data from 3 independent experiments. Box plots show the interquartile range, median (line), and minimum and maximum (whiskers). (**H**) Immunofluorescence analysis of Sprr1a (red) in organoids treated with WT or SFRP1^–/–^ containing EVs or SFRP1^–/–^ containing EVs supplemented with rSFRP1. Nuclei stained with DAPI (blue). Single points represent MFI from 4 single organoids for each biological replicate (*n* = 3–4). (**I**) Real-time qPCR to determine Sprr1a gene expression of organoids treated with WT or SFRP1^–/–^ containing EVs or SFRP1^–/–^ containing EVs supplemented with rSFRP1. Single points represent biological replicates (*n* = 3–4). Statistical analysis by nonparametric Mann-Whitney or Pearson’s correlation testing. *P* values and correlation coefficient indicated in corresponding panels.

To shed light on the mechanism underlying how EV-derived SFRP1 promotes lung fibrosis, we performed bulk RNA-Seq of the lungs with dual exposure to bleomycin and EVs from *Sfrp1*^–/–^ (*Sfrp1*^–/–^ pmLF-EVs) versus *Sfrp1*^+/+^ fibroblasts (*Sfrp1*^+/+^ pmLF-EVs). Overall, 298 genes were significantly increased (Wald’s test *P* < 0.05 and log_2_ fold-change of 0.5) in the *Sfrp1*^–/–^ pmLF-EV group while 236 genes were significantly enriched in the *Sfrp1*^+/+^ pmLF-EV animals (Figure 5A and Supplemental Figure 7A). The *Sfrp1*^+/+^ pmLF-EV group showed increased TGF-β signaling, inflammation, and other fibrosis-related terms based on gene set enrichment analysis for hallmark pathways (Supplemental Figure 7B). We verified activated developmental signaling upon exposure to *Sfrp1*^+/+^ pmLF-EVs compared with *Sfrp1*^–/–^ pmLF-EVs by qPCR (Supplemental Figure 7C). Moreover, our RNA-Seq analysis revealed an increased alveolar differentiation intermediate (ADI) gene signature including keratin 8 in *Sfrp1*^+/+^ pmLF-EV samples compared with the *Sfrp1*^–/–^ pmLF-EV group (Supplemental Figure 7D), suggesting impaired alveolar differentiation as a driver of the fibrotic process (34, 39). In further support, we found a significant correlation between the top genes upregulated in *Sfrp1*^+/+^ pmLF-EV samples (over KO EV) and the ADI gene expression using a published murine lung fibrosis dataset (GSE40151; *r* = 0.67, *P* < 0.001; Figure 5B). Moreover, *Sfrp1* correlated with the expression of ADI-related genes in this dataset (*r* = 0.85, *P* < 0.001, Figure 5C). Notably, cells expressing Sfrp1 surrounded Krt8^+^ cells in areas of active remodeling in fibrotic lungs of mice challenged with bleomycin (Figure 5D). Accordingly, more keratin 8–expressing ADI cells were detected in the lungs of *Sfrp1*^+/+^ pmLF-EV mice compared with *Sfrp1*^–/–^ pmLF-EV mice (Figure 5E). To further validate that SFRP1 affects AT2 differentiation, we cultured pmAT2 with or without recombinant SFRP1 (rSFRP1). SFRP1 induced the accumulation of Krt8^+^ AT2 cells, as assessed by immunostaining (Figure 5F). In addition, pmAT2 cells treated with rSFRP1 transcriptionally converged toward the Krt8^+^ transitional phenotype, upregulating *Krt8* along with other known ADI hallmark genes small proline-rich protein 1A (*Sprr1a*) and integrin beta 6 (*Itgb6*) (Figure 5G). Accordingly, AT2 organoids cultured in the presence of *Sfrp1*^+/+^ pmLF-EVs presented higher expression of the ADI marker *Sprr1a* compared with KO EVs (Figure 5, H and I). In line with previous findings from our group showing that WNT/β-catenin is involved in ADI transition (34), pmAT2 cultured in the presence of rSFRP1 also exhibited increased *Axin2* and *Nkd1*, both known markers of the WNT/β-catenin–dependent signaling (Supplemental Figure 8). To test whether EV-bound SFRP1 also affects WNT/β-catenin–dependent signaling, we used a WNT/β-catenin reporter cell line and indeed observed increased WNT/β-catenin activity of SFRP1 upon *Sfrp1*^+/+^ over *Sfrp1*^–/–^ pmLF-EVs (Supplemental Figure 9A). To investigate if this effect is conserved in primary epithelial cells, we profiled the expression of a WNT/β-catenin target gene in organoids and found an increase in *Axin2* in organoids treated with *Sfrp1*^+/+^ pmLF-EVs over *Sfrp1*^–/–^ pmLF-EVs (Supplemental Figure 9, B and C). Collectively, these data support the notion that EVs secreted by *Sfrp1*-expressing fibroblasts aggravate fibrosis because of the activation of WNT/β-catenin and the ADI AT2 cell phenotype.

### SFRP1 expression is enhanced in IPF and enriched on fibroblast-derived EVs.

**Figure 6:**
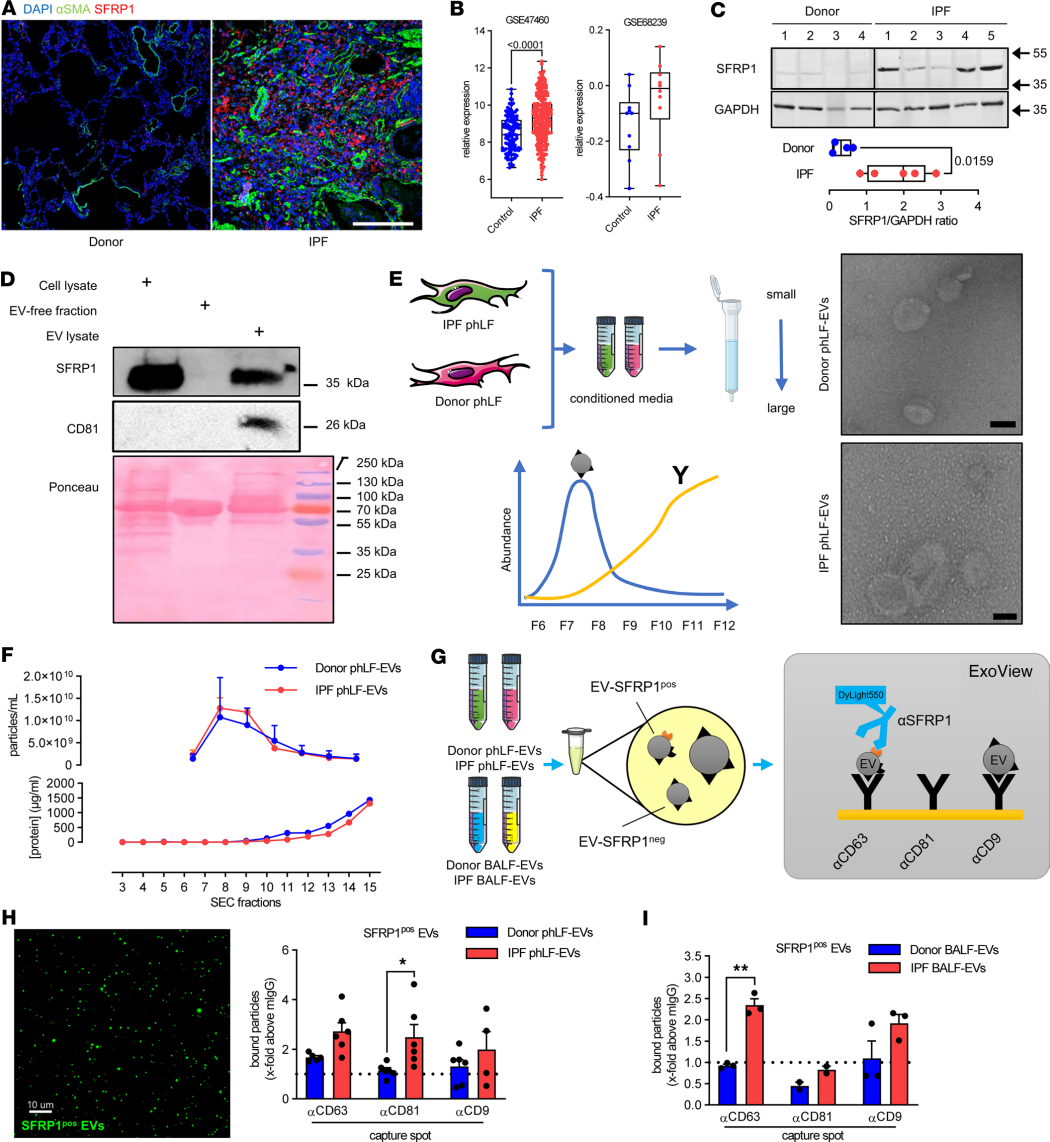
SFRP1 in overexpressed in IPF and is transported by fibroblast EVs. (**A**) Immunofluorescence staining for α-SMA (green) and SFRP1 (red) in FFPE lung sections from patients with IPF or donors. DAPI stains nuclei (blue). Scale bars = 200 μm. (**B**) Gene expression of *SFRP1* in lung tissue from patients with IPF or controls. Data from GSE47460 and GSE68239. Box plots show the interquartile range, median (line), and minimum and maximum (whiskers). (**C**) SFRP1 expression by Western blot on lung tissue from patients with IPF (*n* = 5) or donors (*n* = 4). GAPDH serves as loading control. Densitometry over GAPDH is shown. (**D**) phLFs were cultured and EVs isolated from the cell culture SN. SFRP1 expression in cell lysate, EV-free fraction, and EV fractions. CD81 used as EV-enriched protein and Ponceau shows total protein amount. (**E** and **F**) phLF-EVs were isolated by SEC from conditioned media of control or IPF cells. Expected quantifications for EVs (blue) and proteins (yellow) are shown. Representative electron microscopy of pooled fractions 7–9 (**E**). Particle concentration in fractions 3–16 were quantified by nFCM (**F**). (**G**) Schematic presentation of ExoView analysis workflow for SFRP1 surface expression on EVs. ExoView chips have spotted capture antibodies targeting CD63, CD81, and CD9. EVs bound to the chip via the capture antibodies were visualized using a DyLink 550–conjugated α-SFRP1 antibody. (**H**) SFRP1^+^ EVs from phLFs (donor and IPF) were quantified using the ExoView system. Bound particles positive for SFRP1 at the different capture spots are presented as x-fold above mIgG background. SFRP1-positive EVs in green. Scale bar = 10 μm. (**I**) SFRP1^+^ EVs from human BALF (donor and IPF) were quantified using ExoView. Bound particles positive for SFRP1 at the different capture spots are presented as x-fold above murine IgG background. BALF from 5 donors each were pooled. Statistical analysis by parametric 2-tailed unpaired *t* test (**C**) or 2-way ANOVA (**H** and **I**). *P* values are indicated and *P < 0.05, **P < 0.01.

We next investigated whether EV-bound SFRP1 can be found in human pulmonary fibrosis. We found that SFRP1 is expressed in IPF tissue, particularly in areas of dense fibrosis characterized by α-SMA staining (Figure 6A). Moreover, SFRP1 expression was increased at transcript (Figure 6B) and protein levels (Figure 6C) in IPF lung tissue compared with nondiseased donor tissue. We next were wondering whether SFRP1 is also expressed in EVs from human fibroblasts. To this end, we purified EVs from primary human lung fibroblasts (phLFs) via differential ultracentrifugation as previously published by our group (17, 24) and found SFRP1 to be enriched in vesicle preparations obtained from phLFs (Figure 6D). To further corroborate, we applied size-exclusion chromatography (SEC) and purified EVs from conditioned media of phLFs isolated from IPF tissue or nondiseased donor tissue. We found similar numbers of EVs using electron microscopy and nano–flow cytometry (nFCM) (Figure 6, E and F). Here, we identified EVs in SEC fractions 7–10 by nFCM while proteins were eluted in later fractions. This was verified by a Western blot of the different SEC fractions, which showed a signal for the EV-enriched protein CD63 in fractions 7–10 (Supplemental Figure 10). These EV corresponding fractions also contained SFRP1. In addition to SFRP1’s presence in EVs, we also detected it in CD63-negative fractions, meaning that SFRP1 was secreted via vesicles and as a soluble protein. To verify that SFRP1 was indeed associated with EVs, we performed an ExoView analysis using a chip spotted with CD63-, CD81-, or CD9-specific antibodies and an SFRP1 detection antibody (Figure 6G). This detected EVs carrying SFRP1 from both donor and IPF phLFs and an overall increased secretion of SFRP1^+^ vesicles by IPF phLFs (Figure 6H). SFRP1 was found to be present at the surface of EVs regardless of which tetraspanin was expressed. However, only SFRP1^+^CD81^+^ EVs were statistically significantly increased in phLFs isolated from patients with IPF compared with donors (Figure 6H). Finally, we analyzed BALF from patients with IPF or donors on the ExoView platform. We found SFRP1 was significantly enriched in CD63-harboring EVs from patients with IPF compared with controls (Figure 6I). This verified increased abundance of SFRP1 on the surface of EVs from patients with IPF.

## Discussion

In recent years, emerging evidence suggested a prominent role for impaired cellular crosstalk during fibrosis (40, 41). Consequently, the study of EVs including exosomes has gained significant attention in the field (42). These secreted vesicles are effective mediators of cell-to-cell communication and participate in physiological and pathological processes. Due to their accumulation in body fluids and their ability to participate in (cross-organ) disease mechanisms, EVs are regarded as interesting targets to develop novel therapies and as diagnostic/prognostic biomarkers (43). We and others demonstrated increased numbers of EVs in pulmonary fibrosis (17–19, 44). These vesicles accumulate within the lungs of patients with IPF and can be detected in several body fluids such as BALF, sputum, or urine (17, 18, 45). Previous studies suggest that EVs impact the cellular mechanisms of fibrosis (17, 19, 28, 45, 46); however, the cargo of EVs and their potential functional effect on these pathomechanisms remain poorly understood. Here, we profiled the EV proteome of healthy and fibrotic EVs, used multiomic analysis to unbiasedly identify major cellular sources of EVs during fibrosis, and identified SFRP1 secreted on EVs by fibroblasts as a contributor to lung fibrosis in vivo.

Using the well-established model of bleomycin-induced pulmonary fibrosis, we provide a longitudinal time course of EV secretion during fibrosis initiation, progression, and resolution. We found that EVs are highly enriched during active fibrosis compared with the inflammatory or resolution phases. We therefore used the time point of active fibrosis to delineate the specific proteomic cargos conveyed by fibrotic EVs. For a robust analysis of EVs and their functional effects, the methodology of vesicle isolation is crucial (47). To carry out the proteomic analysis of the vesicles, we applied a well-established and widely used protocol based on differential ultracentrifugation (17, 19, 24). As an additional separation step, we implemented SEC before mass spectrometry–based characterization of isolated EVs, as previously described (48). This step results in elimination of components smaller than 30 nm, such as protein aggregates. Assessment via NTA, TEM, and proteomics validated successful EV isolation, similar to previous reports (17). Our proteomic data further validated our approach, as we observed an enrichment of canonical EV proteins (top 100 listed in Vesiclepedia), such as CD9, CD81, and flotillin-1 (49), in all EV fractions. Notably, we compared all EV fractions with the paired EV-free fraction, thus enabling the identification of proteins transported by fibrotic EVs specifically (i.e., not on control vesicles and not on the EV-free fraction). This approach allowed us to discriminate between proteins secreted during fibrosis and proteins specifically transported by EVs during disease. Of note, we observed that several proteins are secreted in EVs and also not linked to EVs after bleomycin injury. These proteins forming the identified cluster A are relevant to fibrosis and are linked to TGF-β signaling and extracellular matrix. Interestingly, several proteins were primarily found as components of EVs but not in the EV-free fraction, which points to specific cellular mechanisms involved in protein secretion via EVs, with the effect that these proteins a) are packed together with additional vesicular cargo, which might lead to potentiation of the biological effect (9, 50, 51) and b) have a different signaling range, with EVs being able to mediate long-range and even cross-organ effects (52, 53). Our proteomic approach has some limitations that are important to consider: first, while we found several well-known and novel fibrosis markers in our proteomic dataset, some of the proteins previously reported were not detected (54, 55). We have previously described that EVs from fibroblasts carry increased amounts of the WNT protein WNT5A in lung fibrosis (17); however, we did not detect WNT proteins in our proteomic analysis. This could be due to the potential loss of lipid-linked proteins, which is a well-known limitation of such proteomic approaches (56, 57). Here, we report the presence of SFRP1 in fibrotic EVs. We further validated the presence of SFRP1 on EVs by additional EV separation techniques, including SEC, which verified the presence of SFRP1 in EV-containing fractions. The single-vesicle analysis using ExoView demonstrated the occurrence of SFRP1 at the vesicle surface. Second, we focused our proteomic analysis on the day 14 time point. Our initial time course shows that EV secretion is dynamic, and future studies are needed to delineate the temporal change in composition and delineate the most likely different source of EVs throughout inflammation, fibrosis, and resolution.

A key question in the field is the cellular origin of EVs, and to address this question, we applied a multiomic approach integrating our proteomic dataset with published scRNA-Seq data from our group (34). Indeed, we found that BALF-derived EVs contain distinct protein signatures associated with several cell types in the lung. Remarkably, we detected proteins expressed by all cell types in nondisease control samples, while proteins specific to fibrotic EVs were primarily linked to fibroblasts and alveolar epithelial cells. EVs secreted by epithelial cells and immune cells have also been reported to contribute to lung diseases (19, 58–61). In our analysis fibroblasts stood out as a significant source of EVs, which is consistent with our previous report on increased EVs in IPF (17). Further, our proteome analysis revealed a high enrichment of SFRP1 in fibrotic EVs. Recently, we demonstrated by a detailed immunofluorescence analysis of micro-CT staged IPF patient tissues that SFRP1-expressing fibroblasts are present in both mildly affected and fibrotic dense areas (36). Using extensive scRNA-Seq analyses, we and others identified SFRP1 as a key marker of pathological transitional fibroblasts, which precede TGF-β1–induced transition into extracellular matrix–producing myofibroblasts (36, 38). Consequently, the SFRP1-positive fibroblast subpopulation contributes to a profibrotic milieu within the IPF lung (35–37). In line with this, we provide here evidence that SFRP1 is a critical mediator of the profibrotic function of fibroblast-derived vesicles in lung fibrosis. It is further notable that several recent reports highlight that fibroblasts utilize EV secretion to exert autocrine and paracrine effects that contribute to fibrosis (13, 62). We showed that EVs contribute to autocrine effects in human fibroblasts in IPF (17). Similarly, Chanda et al. reported that senescent fibroblasts secrete EVs and thus promote fibroblast invasion (14). In addition, paracrine effects of fibroblast-derived EVs on lung epithelial cells have been demonstrated,with EVs from IPF lung fibroblasts inducing senescence in lung epithelial cells in vitro (63). Vice versa, fibroblasts are responsive to EVs secreted by other cells, such as immune cells (64), or lung epithelial cells, which impact TGF-β/WNT crosstalk in fibroblasts (65). Here, we expand on the paracrine effect of fibroblast-derived EVs and their effect on AT2 cell reprogramming as a key feature of lung fibrosis (66, 67). We demonstrate that fibrotic EVs impair AT2 stem cell function using a well-established organoid assay. Moreover, we found that SFRP1 leads to an increase in Krt8^+^ ADI cells, which have recently been identified in both experimental and human fibrosis (34, 39). Importantly, when using SFRP1 KO EVs, we found reduced Krt8^+^ ADI gene expression in organoids and in vivo along with attenuated lung fibrosis in vivo. Recently, Wang et al. demonstrated that Krt8-KO mice are protected from experimental fibrosis and that Krt8^+^ cells activate fibroblasts, which highlights the pathological feed-forward loop between fibroblasts and alveolar epithelial cells, potentially mediated by EVs carrying SFRP1. Together, these data support the notion that fibroblast-derived EVs are key components of impaired cellular crosstalk in fibrosis and further uncover the diversity of the EVs with regard to source, composition, and functional outcome. Another source of EVs connected to fibrosis is mesenchymal stromal cells (MSCs), which secrete vesicles exhibiting antifibrotic properties. EVs derived from these cells are suggested as a potential cell-free therapy (68, 69). Intratracheal instillation of exogenous MSC-EVs attenuated established fibrosis (70), raising the question how the EV cargo from these cells differs from other sources. Future studies are needed to further explore these differences, and future technological advancements will aid in determining specific markers on EVs’ surface (71, 72).

Our study provides evidence that fibrotic EVs derived from BALF are sufficient to initiate profibrotic mechanisms in multicellular ex vivo models. We have previously shown that fibrotic EVs enhanced collagen accumulation within the lung in vivo; however, these profibrotic effects were only observed after mild injury was induced by bleomycin (19). Similarly, here we transferred pmLF-derived EVs to mice injured with low-dose bleomycin, which might already cause SFRP1 expression and thus confounds any effects of EVs. Although we used WT C57BL/6 mice, we were able to show that the exacerbation of fibrosis seen upon Sfrp1^+/+^ pmLF-EV injection is lost after injection with Sfrp1^–/–^ pmLF-EVs. There are several potential reasons that we do not observe induction of fibrosis in otherwise healthy lung tissue in vivo as we observed ex vivo, including insufficient number/dosage of EVs and the different sources (BALF- versus fibroblast-derived EVs) and as such likely different composition of EVs in our current study. One can also speculate that homing of monocyte-derived macrophages, which is lacking in the ex vivo system, might contribute to clearing EVs and thus dampen the potential profibrotic effects in vivo. These cells further contribute to the activation of TGF-β signaling, which was reduced in our in vivo experiments but not affected ex vivo.

Here, we found fibroblast-derived SFRP1 to be a highly expressed protein cargo of fibrotic EVs. Notably, our data demonstrate that SFRP1 in fibrotic EVs is required, at least partially, for the pro-fibrotic effect of these vesicles on lung fibrosis in vivo. EVs derived from *Sfrp1*-deficient fibroblasts did not exacerbate bleomycin-induced lung fibrosis in vivo contrary to vesicles from WT fibroblasts. Mice deficient for *Sfrp1* exhibit enhanced kidney fibrosis after unilateral ureteral obstruction compared with WT mice (73). In the lung, however, global *Sfrp1*-KO animals did not show differences in fibrosis development after exposition to bleomycin (74). The conflicting results might be explained by redundancy and adaptation. SFRPs constitute a family of proteins, which have redundant roles, and the study on kidney fibrosis suggests that SFRP1 might have a different organ-specific role. In addition, the *Sfrp1*-deficient mice used were whole-body–knockout animals, and adaptive responses during lung development and homeostasis are likely. SFRPs are primarily described to modify WNT signaling (75). The exact mechanism of action of SFRP1 on the WNT signaling is highly dependent on the cellular context and SFRP concentration and has been reported to activate WNT/β-catenin signaling at low concentrations and to reduce it at higher concentration (76). Intriguingly, we found that SFRP1 on EVs activated WNT/β-catenin pathway compared with EVs lacking SFRP1. These data validate a previous observation that EV-linked SFRPs promote WNT/β-catenin signaling (77). Notably, SFRP1 secreted via EVs can enter cells and modulate β-catenin activity in the nucleus (76, 77). It is also of interest that nonvesicular SFRPs have been described to promote the resecretion of WNT ligands within EVs (78), which could further increase WNT/β-catenin signaling in lung fibrosis (27). Our data further support a distinct role for vesicular SFRP1 compared with nonvesicular SFRP1, which needs to be further studied in the future.

The dissection of EV cargo has largely focused on the microRNA (miR) content within these vesicles (12, 18–20, 45, 46, 65). Here, we provide an in-depth analysis of the EV proteome with 774 proteins identified in healthy and fibrotic EVs. Interestingly, SFRP1 protein expression is known to be regulated via miR (79, 80). It will be exciting in future studies to systematically compare protein and miR cargo of the same EVs to identify potential common downstream mechanisms and pathways that are targeted in effector cells. This comparison could reveal novel therapeutic approaches, both targeting EV cargo together as well as identifying the key downstream effects to intervene with in a pathophysiological setting. Importantly, we further demonstrate fibroblast-derived vesicular SFRP1 can also be found in human biosamples. We found detectable levels of vesicular SFRP1 in BALF from patients with IPF, which thus might serve as a potential biomarker. To this end, using the ExoView platform, we detected EVs and their cargo in small volumes of bodily fluids, specifically in BALF, without the need to isolate EVs and thus representing a viable option for clinical implementation. Future studies investigating SFRP1 in other compartments and in bodily fluids that are routinely collected, such as blood/plasma and/or urine, where EVs have already been detected in cohorts with pulmonary fibrosis (45), will be crucial to further evaluate its biomarker potential. In summary, our work reveals a highly altered EV protein cargo promoting lung fibrogenesis and identifies fibroblast-derived SFRP1 on EVs as a potential therapeutic target and biomarker for IPF.

## Methods

### Sex as a biological variable

Only male animals were used for this study, as IPF has a higher prevalence in males compared with females in humans and male mice develop more progressive fibrosis following bleomycin exposure. We expect our findings to be relevant to both males and females.

### EV concentration

EVs were concentrated from cell-free BALF and culture media under sterile conditions by differential ultracentrifugation using an already described protocol (17, 19, 24) or SEC (see below). In brief, samples were first centrifuged at 600*g*, 10 minutes, 4°C, to remove cells and cellular debris. Then, the supernatant was subjected to a first centrifugation at 10,000*g*, 30 minutes, 4°C, to pellet large vesicles and apoptotic bodies. The corresponding supernatant was centrifuged at 110,000*g* for 2.5 hours at 4°C to pellet EVs. At this step, the supernatant (termed here EV-free SN) was saved and concentrated by Amicon Ultra-0.5 centrifugal 10,000 MWCO filters (Merck-Millipore). The pelleted EVs were washed in cold, 0.1 μm filtered 1× PBS and centrifuged at 110,000*g*, 2.5 hours, 4°C. Finally, EVs were resuspended in 200 μL of 0.1 μm filtered 1× PBS. In this study, we have used 2 separate ultracentrifugation settings: Thermo Fisher Scientific Sorval WX Ultra 90 ultracentrifuge with T-647.5 and TFT-80.2 fixed-angle rotors (for characterization and proteomics) or Beckman Coulter L-80 ultracentrifuge with Type 45 and Type 50.4 2 fixed-angle rotors (for functional testing). Before sending out for label-free proteomics, EVs underwent SEC with a cutoff of 30 nm, according to the manufacturer’s instruction (Exo-spin, Cell Guidance Systems).

For phLF media, the samples were centrifuged twice (300*g* for 5 minutes and 4,000*g* for 15 minutes) to pellet dead cells and larger cell debris, respectively. The obtained supernatant was concentrated to 500 μL by repeated ultrafiltration at room temperature (RT) (Amicon Ultra Centrifugal Filter, 100 kDa MWCO, Merck-Millipore) at 4,000*g*, 15 minutes, and loaded on qEVoriginal/70 nm Gen 2 SEC columns (IZON Science Ltd) prewashed with 10 mL of 0.1 μm filtered Dulbecco’s PBS following the manufacturer’s instructions. Vesicles were eluted using Dulbecco’s PBS, and 24 fractions of 500 μL were obtained. The protein concentration of the 24 fractions was determined by utilizing the Pierce BCA protein assay kit following the manufacturer’s instructions (Thermo Fisher Scientific). Count and concentration of EVs were analyzed by nFCM (NanoFCM Co., Ltd.). Finally, EV-containing fractions (7–9) as detected by nFCM were pooled and used for subsequent experiments.

### Proteomic analysis

EVs concentrated using differential ultracentrifugation were resuspended in Triton X-100–based lysis buffer (HEPES 50 mM pH 7.4, NaCl 150 mM, EDTA 5 mM, Triton X-100 0.5%), and protein amount was measured using a Lowry-based method (DC Protein Assay Kit 5000111, Bio-Rad). A total of 10 μg protein per sample was subjected to protein digestion and peptide purification using the iST label-free sample preparation kit (PreOmics GmbH). From the EV-free supernatant, proteins were precipitated overnight at –20°C after mixing the supernatant with ice-cold acetone (1:4). Precipitated proteins were pelleted at 4,000*g* at 4°C for 10 minutes; the supernatant was discarded and the protein pellet air-dried for 10 minutes. Proteins were resuspended in 6 M guanidinium hydrochloride buffer [6 M guanidinium hydrochloride, 100 mM Tris-HCl pH 8, 10 mM tris(2-carboxyethyl)phosphine, 50 mM circulating anodic antigen], incubated at 95°C shaking for 10 minutes, cooled down, and sonicated for 10 cycles at the Bioruptor (30-second pulse + 30-second pause). Protein concentration was measured and 20 μg was diluted 1:10 with digestion buffer (10% acetonitrile, 25 mM Tris) and digested with Trypsin/LysC 1:50 (enzyme/protein) overnight at 37°C. Samples were acidified to 1% trifluoroacetic acid and peptides enriched using SDS-RPS stage tips as previously described (81). Approximately 1 μg of peptides was separated in 1-hour gradients with reverse-phase chromatography being performed with an EASY-nLC 1000 ultrahigh-pressure system (Thermo Fisher Scientific), which was coupled to a Q Exactive Mass Spectrometer (Thermo Fisher Scientific) as previously described (82).

### Statistics

All data are expressed as mean ± SD and analyzed with GraphPad Prism 8 software. Normal distribution of the data was determined by Kolmogorov-Smirnov testing with Lilliefors’ correction before applying a parametric test. Two-tailed unpaired *t* test was used when values followed normal distribution. Two-tailed nonparametric Mann-Whitney test was used with comparison between groups when data did not follow a normal distribution. For comparison of more than 2 groups, 1-way ANOVA was used followed by Dunnett’s post hoc test. For correlation study, nonparametric Spearman’s test or Pearson’s was used, depending on the distribution of the data. For each comparison, 2-tailed *P* value is indicated. A *P* value less than 0.05 was considered significant.

### Study approval

#### Patient-derived samples.

Primary human fibroblasts and pulmonary tissue from patients with IPF and donors (patients without diagnosed chronic lung disease) were obtained from the CPC-M bioArchive at the CPC (Munich, Germany), the biobank at the UGMLC, and the University of Pittsburgh. The studies were approved by the local ethics committees of the Ludwig-Maximilians-University (Munich, Germany) (ethic vote 333-10), the Justus-Liebig-University Giessen (ethic votes 58/15 and 111/08), the Philipps-University Marburg (ethic vote #23-201 BO), and the University of Pittsburgh’s Institutional Review Board (IRB PRO14010265). Written informed consent was obtained for all study participants.

#### Animal studies.

The procedures involving animals in this study have been approved by the institutional animal care and use committee of the University of Colorado Denver, the ethics committee of the Helmholtz Zentrum München and the Regierung von Oberbayern (Munich, Germany), and the “Comité d’Ethique de l’Expérimentation Animale du grand campus” of the University of Burgundy (Dijon, France) and the “Ministère de l’Enseignement Supérieur, de la Recherche et de l’Innovation” (Paris, France) under the project references 115517(04)1E, AZ 55.2-1-54-2532.130.2014, and APAFIS #26877.

### Data availability

Data presented in this manuscript, including values for all data points shown in graphs, are available in the Supporting Data Values supplemental file or from the corresponding authors upon request. Bulk RNA-Seq data have been deposited to the GEO database (GSE272679).

Detailed description of further methods is provided in the Supplemental Methods linked to this manuscript.

## Author contributions

OB, DS, EFP, BB, AS, YS, QH, MCMN, EJ, JCP, M Mozurak, AEB, UO, ALJ, and M Lehmann designed and performed experiments, analyzed data, and prepared figures; CHM performed mass spectrometry experiments and analysis as well as integrative analysis of proteomic data with scRNA-Seq data and prepared figures; OB, M Lehmann, G Burgstaller, and MK designed experiments and oversaw all data analysis; HBS supervised proteomic data analysis and experimental design; CC contributed to nanoparticle-tracking experiments and analysis; TH performed TEM; TP and PC provided technical assistance and important intellectual content; M Lindner, AH, CR, and AG collected and provided human tissue samples; M Mann, AÖY, and OE analyzed and interpreted results and brought important intellectual content; OB, G Bange, BTS, AÖY, HBS, M Lehmann, G Burgstaller, and MK provided resources and funding; and OB, M Lehmann, G Burgstaller, and MK drafted the manuscript. All authors have critically revised the manuscript. All authors have read, reviewed, and approved the final manuscript as submitted to take public responsibility for it.

## Supplementary Material

Supplemental data

Unedited blot and gel images

Supporting data values

## Figures and Tables

**Table 2:**
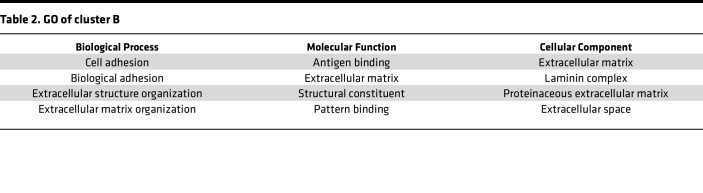
GO of cluster B

**Table 1:**
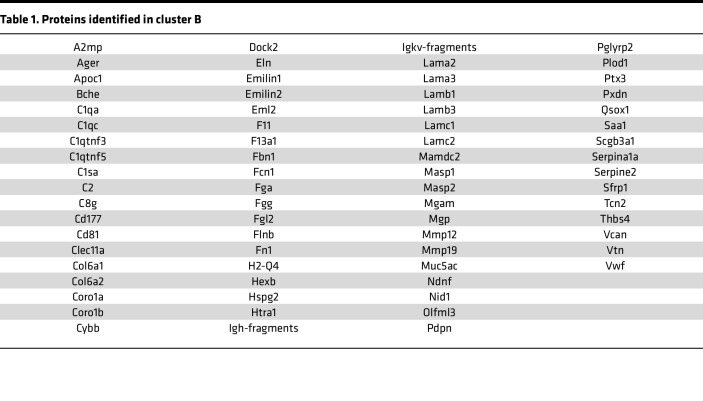
Proteins identified in cluster B

## Version 1 09/24/2024

Electronic publication
