# Supplementary material for: Fibroblast-derived extracellular vesicles contain SFRP1 and mediate pulmonary fibrosis
Source: JCI Insight. 2024 Aug 15;9(18):e168889. doi: 10.1172/jci.insight.168889 (PMC11457858; doi:10.1172/jci.insight.168889)
Supplement: Supplemental data [file jciinsight-9-168889-s187.pdf]

Online Supplemental Material

Fibroblast-derived extracellular vesicles contain SFRP1 and mediate pulmonary fibrosis

Olivier Burgy, Christoph H. Mayr, Déborah Schenesse, Efthymios Fousekis Papakonstantinou, Beatriz Ballester, Arunima Sengupta, Yixin She, Qiangjiang Hu, Maria Camila Melo-Narvaéz, Eshita Jain, Jeanine C. Pestoni, Molly Mozurak, Adriana Estrada-Bernal, Ugochi Onwuka, Christina Coughlan, Tanyalak Parimon, Peter Chen, Thomas Heimerl, Gert Bange, Bernd T. Schmeck, Michael Lindner, Anne Hilgendorff, Clemens Ruppert, Andreas Günther, Matthias Mann, Ali Önder Yildirim, Oliver Eickelberg, Anna Lena Jung, Herbert B. Schiller, Mareike Lehmann, Gerald Burgstaller, Melanie Königshoff

List of Supplementary Material:

Supplemental Methods

Figure S1. Specific set of proteins are enriched in fibrotic EVs.

Figure S2. GO annotations of the protein clusters.

Figure S3. Proteins enriched in fibrotic EVs are dysregulated during pulmonary fibrosis.

Figure S4. Proteins linked to fibrotic EVs are expressed by mesenchymal cells.

Figure S5. The fibrotic EV protein SFRP1 is mainly expressed by pathological transitional fibroblasts.

Figure S6. SFRP1 is the SFRP isoform the most expressed in fibroblasts.

Figure S7. Fibrosis-related mechanisms are increased in WT-EVs animals.

Figure S8. SFRP1 promotes WNT/β-catenin signaling in AT2 cells.

Figure S9. SFRP1 KO-EVs inhibits WNT/β-catenin and TGF-β signaling.

Figure S10. SFRP1 is secreted both in CD63 positive EVs and free.

Table S1. Primers used in this study.

Supplemental Methods

Animals

8-week-old C57Bl/6J mice were purchased from the Jackson Laboratory (Bar Harbor, ME, USA) or Charles River (Saint-Germain-sur-l'Arbresle, France). Only male animals were used for this study, as IPF has a higher prevalence in males compared to females in human and as male mice develop a more progressive fibrosis consecutive to bleomycin exposition. *S*ecreted frizzled-related protein 1 (*Sfrp1*) deficient mice SFRP1-2AKI (ref MGI:3796251) (1) were ordered from MRC Harwell, Mary Lyon Centre, Harwell Campus, Oxfordshire. Here, the SFRP1 is a targeted (null) knock-out by deletion of exon1 by replacing it with a nuclear-localized lacZ KI cassette (IRES-T-LacZ-bpA) and a PGKneobpA (1700 bp) lox. The lacZ-neo cassette replaces exon 1 of the *Sfrp1* gene, abolishing gene function. The SFRP1-2AKI were received as frozen embryos (Stock ID: FESA:1734 Sales order reference: RQ217) and embryo transfer (FET) was performed in C57Bl/6J. The SFRP2 deletion was finally backcrossed, so that all progeny was SFRP2 homozygous wild type (+/+) for SFRP2. All the animals were housed in pathogen-free conditions, with environmental enrichment, in rooms maintained at constant temperature and humidity under a 12-h light cycle, and had access to food and water *ad libitum*.

Experimental lung fibrosis mouse model

Mice were anesthetized with isoflurane and underwent orotracheal aspiration of bleomycin (Willow Birch Pharma, Taylor, MS, USA) with a single dose of 2.5 U/kg. Control mice were injected with sterile 0.9% NaCl. At the indicated time points, mice were anesthetized with a mixture of ketamine/xylazine before lung function testing (FX2-Flexivent®, SCIREQ, Montreal, QC, Canada). Three readings were taken for each animal. After exsanguination via the *vena cava*, broncho-alveolar lavage fluid (BALF) was collected, centrifuged (600g, 15min, +4°C) and cell-free BALF were stored at -80°C before being processed. Lung tissue were then

harvested and either fixed with formalin for histology or flushed with 1X PBS and snap frozen in liquid nitrogen for RNA or protein extraction.

Generation of murine PCLS

Precision cut lung slices (PCLS) were generated from healthy C57Bl/6J mice as previously described (2). Briefly, using a syringe pump, the mouse lungs were filled via a tracheal cannula with 1.5% (w/v) warm, low gelling temperature agarose (Merck-Sigma Aldrich, Saint-Quentin-Fallavier, France) in sterile DMEM medium (Dutscher, Bernolsheim, France), supplemented with 1% penicillin/streptomycin/amphotericin B (Dutscher). Afterwards, the lungs were removed and transferred on ice in cultivation medium for 10min to allow the agarose to solidify. Each lung lobe was separated and cut with a VT1200S vibratome (Leica, Nanterre, France) at 300µm thickness to generate 4mm diameter punches. PCLS were cultivated in DMEM supplemented with 0.1% FBS (Dutscher) and antibiotics. 24h after slicing, PCLS were exposed to EVs (BALF-EVs from mice with bleomycin-induced lung fibrosis or control mice. 1:1000 cell to vesicle ratio) for seven additional days. Media with EVs was replenished every 72h. Tissue viability was confirmed by WST-1 assay (Merck-Sigma Aldrich) as described (2).

Primary murine cell isolates

Primary alveolar type 2 (AT2) cells were isolated as previously described (3, 4) with slight modifications. Mice were anesthetized (ketamine/xylazine) before exsanguination by cutting the vena cava, and the lungs were flushed with PBS. Mouse lungs were intratracheally inflated with dispase (BD Bioscience) followed by 300µl instillation of 1% low gelling temperature agarose (Merck-Sigma Aldrich). Lungs were excised, minced and filtered through 100, 20 and 10µm nylon meshes (Sefar). White blood cells were depleted with CD45, and epithelial cells were selected using CD326 (EpCAM) magnetic beads (respectively 130-052-301 and 130-105-958, Miltenyi Biotec), according to the manufacturer’s instructions. EpCAM+ cells were resuspended in DMEM containing 2mM l-alanyl–L-glutamine dipeptide (Gibco, Thermo Fisher Scientific), 100U/ml Penicillin/Streptomycin (Merck-Sigma Aldrich), 3.6mg/ml glucose

(AppliChem) and 10mM HEPES (Thermo Fisher Scientific) until further use for the organoid experiments. For the experiments with recombinant SFRP1 (rSFRP1), 5 × 10^5 AT2 cells were seeded on coverslip in 24-well plates and cultured for 24h in DMEM (Sigma) supplemented with 2% GlutaMAX^{TM} (Gibco, Thermo Fisher Scientific), 1% penicillin/streptomycin (Gibco, Thermo Fisher Scientific), 3.6 mg/mL glucose (Merck-Sigma Aldrich, G7021), 1% amphotericin B (Merck-Sigma Aldrich, #A2942), and 10mM HEPES (15630-080, Gibco, Thermo Fisher Scientific) and 10% FBS (#S12450H, R&D systems). Then, 24h before the beginning of the experiments, cells underwent two washes with PBS and were then incubated in starvation medium (same culture media as above with 0.1% FBS). At the initiation of the experiment (designated as time zero), the cells were transitioned to starvation medium with or without 100ng/ml rSFRP1 (#5396-SF-025, R&D system). Cells were refreshed with or without 100ng/ml SFRP1 in starvation medium on day 3. Following a total incubation period of 6 days, coverslips were rinsed in PBS, fixed with 4% PFA (#AAJ61899AP, Fisher Scientific) for 15 minutes at RT, and then rinsed twice with PBS before immunostaining.

Primary mouse lung fibroblast (pmLF) isolation was performed as previously described (5). Sfrp1 deficient or control mice were anesthetized (ketamine/xylazine) before exsanguination by cutting the vena cava. After flushing, lung tissue was harvested and dissected lung lobes were placed in ice cold sterile PBS in a 6-well plate. Using sterile scalpels, the lobes were dissected into small 1-2mm pieces and immediately transferred into a 50ml falcon containing collagenase 1 (diluted 5mg per 50µl of 1X PBS). Collagenase digestion was carried on at 37°C at 400rpm for 1 hour and next transferred into a 70µm-filter placed on a fresh falcon tube. Using the head of a syringe pistol the digested lung pieces were scratched onto the filter and rinsed thoroughly with sterile 1X PBS. The final suspension was centrifuged for 5 min at 400rpm at 4°C. Lastly, the supernatant was discarded and the pellet was resuspended in fresh cell culture medium (20% FBS and 100U/ml of penicillin/streptomycin supplemented DMEM/F-12 media) and cultured under standard cell culture conditions. To produce culture media for EV concentration, pmLF were cultivated in 175 cm^2 flasks in DMEM supplemented with EV-

free FBS (System Biosciences, Palo Alto, CA, USA). Importantly, cells were used at passages less than 12 and did not reach more than 90% confluency and viability (Trypan blue, Merck-Sigma Aldrich) was above 95%. Conditioned media from 48h of culture were collected from three independent clones of wild-type or *Sfrp1*$^{-/-}$ pmLF.

Patient-derived lung fibroblasts

Primary human lung fibroblast (phLF) have been isolated from patients with IPF or control patients as previously described (6). For EV generation, the cells were used in passage 6-10 and cultured in three 175 cm² flasks using DMEM/F-12, supplemented with 20% FCS and 1% penicillin/streptomycin. The cells were grown at 37 °C with 5% CO2 until 60–70% confluency. Human cell cultures were then incubated in an EV-depleted starvation medium (DMEM/F-12, 1% FBS (Capricorn Scientific), 1% penicillin/streptomycin) at 37°C with 5% CO2 for 24 hours. The medium was EV-depleted by ultracentrifugation (100 000g, 2h, 4°C). The medium was replaced with fresh starvation medium and incubated for 48 hours. Then, the culture medium was collected, and the cells were washed with HBSS, which was collected together with the medium until EV concentration by a size-exclusion chromatography (SEC) protocol as described below.

Organoid assay

Organoids were cultured as previously described (4, 7, 8) with some modifications. MLg mouse lung fibroblasts (CCL-206, ATCC) were proliferation-inactivated by incubation in culture medium containing 10µg/ml mitomycin C (Merck) for 2h, followed by three washes in warm PBS (Life Technologies), and allowed to recover in culture media for at least 1h; 10,000 EpCAM+ cells suspended in 50µl growth factor-reduced Matrigel (Corning) were diluted 1:1 with 10,000 MLg cells in 50µl DMEM/F12 containing 10% (w/v) FBS and seeded into 96-well imaging plates (Corning). Cultures were maintained in DMEM/F12 containing 5% FBS, 100U/ml penicillin/streptomycin, 2mM L-alanyl–l-glutamine dipeptide (Gibco), 2.5µg/ml amphotericin B (Gibco), 1× insulin-transferrin-selenium (Gibco), 0.025µg/ml recombinant

human EGF (Merck), 0.1µg/ml cholera toxin (Merck-Sigma Aldrich), 30µg/ml bovine pituitary extract (Merck-Sigma Aldrich) and 0.01µM of freshly added all-trans retinoic acid (Merck-Sigma Aldrich) on top of the matrigel; 10µM Y-27632 (Tocris), a Rho-associated kinase inhibitor, was added to the media for the first 48h of culture. EVs (1:1000 epithelial over vesicle ratio) were added to the organoid media from day 0 and in every culture media exchange. Media were refreshed every other day. Microscopy for organoid quantification at day 14 was performed using a microscope Axiovert 40 (Zeiss). Individual organoids with a minimum size of 50µm were manually counted by two investigators in a blinded manner. Organoid forming efficiency was calculated by dividing the number of organoids formed at D14 by the number of epithelial cells plated for the assay.

Extracellular vesicle characterization

EV samples were characterized for size distribution and particle concentration by nanoparticle tracking analysis using a NanoSight NS300 system equipped with a green laser and a software version: NTA 3.2 Dev Build 3.2.16 (Malvern Panalytical, Malvern, UK). Frozen EVs were gently thawed on ice and diluted in a final volume of 500µl of 0.1µm-filtered 1X PBS. Diluted samples were injected using a 1ml syringe at constant flow (25µl/min). Absence of air bubbles in the analysis chamber was confirmed before each measurement. Vesicles were tracked by a high frame rate camera (sCMOS camera, 25 frames per second) over a 30 sec time lapse for a total of 5 replicates. Image acquisition and analysis were performed for all samples at constant temperature (22±0.1°C) with a set viscosity (1.05 cP) as well as fixed camera level (14) and detection threshold (6). Values from the five readings were manually checked to identify potential outliers.

To determine size and concentration of SEC-purifed EVs, nano flow cytometry (nFCM) was performed as recently described (9). In brief, a Nano Analyzer (NanoFCM Co., Ltd, Nottingham, UK) equipped with a 488 nm laser underwent calibration using the quality control beads (250nm beads, NanoFCM Co.) with a defined concentration of 2.08 x 10^8particles/ml,

serving as both calibration and reference for particle concentration. The silica nanosphere bead cocktail (S16M-Exo, NanoFCM Co.) contains particles of four different sizes (68nm, 91nm, 113nm, 135nm) and served as size reference standards to calibrate vesicle size measurements. Background signal analysis was conducted using DPBS/modified (0.1µm sterile filtered, Cytiva). Its signal was subtracted from subsequent measurements. Sample acquisition was performed as recommended by the manufacturer (1 minute, 1kPa). Cell-free human BALF samples were diluted with DPBS/modified (0.1µm filtered, Cytiva) to achieve particle counts within the optimal range of 3,000-12,000 events per minute. Particle concentration and size distribution were determined using nFCM software (NF Profession V2.3).

The expression of EV surface proteins was assessed by using the Exoview R100 platform (NanoView Biosciences, Boston, Massachusetts, USA) in combination with human exosome plasma kits (Unchained Labs, Pleasanton, California, USA). Equal volumes (3.5 µL each) of fraction 7-9 from phLF-EVs or 1:1,000 diluted human BALF were mixed and 50 µL loaded per chip. Three independent biological replicates of isolated phLF-EVs were analyzed. For BALF, equal volumes from five donors each were combined and loaded onto a single chip. Sample incubation occurred overnight at RT, following the manufacturer's recommendations for chip handling. For surface marker immuno-fluorescence staining, rabbit anti-SFRP1 (Lot 1018831-3, Abcam) was labeled by using DyLight550 conjugation Kit (fast) – Lightning-Link (Abcam) and used at a concentration of 1.5µg/mL. Antibody incubation was performed for 1h at RT. The immunostained chips were washed and dried. Imaging and data acquisition were performed with the ExoView R100 platform. Data analysis was conducted with the ExoView Analyzer 3.2 (NanoView Biosciences).

Electron microscopy

For murine BALF-EVs, vesicles were diluted in 1X PBS and collected in carbon-coated copper grids, then the samples were stained with uranyl formate and imaged using a ThermoFischer

Tecnai G2 12 BioTwin equipped with a RX80(8Mpix) AMT camera (Electron microscopy core, Anschutz Medical Campus, University of Colorado). For EVs from phLFs, 5µl of vesicle suspensions (from SEC fractions 7-9) were applied to hydrophilized carbon coated copper grids. After blotting and a washing step with a droplet of double distilled water samples were stained with 2% uranyl acetate solution. Grids were analyzed using a JEOL JEM-2100 transmission electron microscope operating at 120 kV. Images were acquired with a F214 fast-scan CCD camera (TVIPS, Gauting).

Western blot

Cells, lung homogenates and EV-pellets concentrated by differential ultracentrifugation were lysed in Triton X-100 lysis buffer supplemented with protease and phosphatase inhibitors (Roche Diagnostics, Mannheim, Germany) and protein concentrations were quantified using a modified Lowry assay (Bio-Rad). Samples were concentrated if necessary, by Amicon Ultra-0.5 centrifugal 10,000 MWCO filters (Merck-Millipore). Reducing conditions (4x Laemmli loading buffer: 150 mM Tris HCl, 275 mM SDS, 400 mM dithiothreitol, 3.5% (w/v) glycerol, 0.02% bromophenol blue) were used. Samples were loaded into 10-12% SDS-PAGE gel and transferred to a nitrocellulose membrane that was blocked afterwards in PBS supplemented with 0.1% Tween20 (PBS-T) and 8% non-fat milk. Blocked membranes were incubated with primary antibodies directed against TSG101 (HPA006161, Sigma Aldrich), Nidogen-1 (ab14511, Abcam), SFRP1 (ab126613, Abcam), α-SMA (ab5694, Abcam), AGER (ab3611, Abcam), QSOX (LS-C802615, Life Span), EGFR (4267, Cell Signaling), HSC70 (sc-7298, Santa Cruz Biotechnology), β-actin (A5316, Sigma) and GAPDH (sc-47724, Santa Cruz Biotechnology) at dilution of 1:1000 in PBS-T + 5% non-fat milk. Finally, membranes were incubated with Peroxidase-AffiniPure Goat anti-rabbit and anti-mouse IgG (Jackson ImmunoResearch, UK). The signal was detected by enhanced chemiluminescence reagents (Immobilon Crescendo Western HRP substrate, Merck Millipore) and was imaged with a ChemiDoc MP Imaging System (Biorad). Ponceau S staining (Merck-Sigma Aldrich), HSC70, β-actin and GAPDH served as loading controls.

For SEC-purified EVs, samples from fractions 6 to 12 (50µl each) were collected and used for western blot. To each tube, 12.5µL of 5x Laemmli buffer with or without β-mercaptoethanol was added and proteins denatured at 95°C for 5 minutes. Samples were then loaded on a 12.5% SDS gel, separated in Tris-Glycine SDS Running Buffer and blotted via wet blot with Towbin buffer on a nitrocellulose membrane. Membranes were blocked in a solution of TBST (TBS with 0.1% Tween®20), containing 10% skim milk powder and 5% BSA, for 1 hour at RT. Primary antibodies (rabbit anti-SFRP1, Lot 1018831-3, 1:1000, abcam; and mouse anti-CD63, Lot #E1711, 1:500, Santa Cruz Biotechnology) were diluted in TBST containing 4% BSA. SFRP1 was detected in samples prepared under reducing conditions, while CD63 was detected in the other set of samples with non-reducing conditions. The incubation was performed overnight at 4°C. On the next day the secondary antibodies (anti-rabbit, Lot L27A9, 1:2000, Cell Signaling Technology; and anti-mouse, Lot #J2621, 1:2000, Santa Cruz Biotechnology) were incubated for 1.5 hours at RT. ECL detection was prepared with a western blotting detection reagent (Amersham ECL Prime Western blotting Detection Reagent, Cytia) and was performed using a Chemiluminescence Imager (Advanced Fluorescence and ECL Imager, Intas Science Imaging Instruments, Göttingen, Germany) to visualize protein expression. To analyze the surface protein expression of SFRP1 on EVs, fractions 1-12 from SEC were used. For this, 10µL of each fraction was applied onto a nitrocellulose membrane (Amersham Protran, cytiva, Freiburg, Germany). Membranes were blocked, incubated with antibodies and detected as described for Western Blots.

ELISA assay

Levels of Advanced Glycosylation End-Product Specific Receptor and Epidermal Growth Factor Receptor were determined using commercial ELISA kits (MRG00 and MEGFR0, both from R&D Systems, Minneapolis, MN, USA) according to the recommendations of the manufacturer. Data are presented as target concentration normalized to 2 x 10^8 vesicles.

Quantitative PCR

RNA was extracted from PCLS using an adapted version of the ZYMO quick-RNA protocol (Zymo Research, Orange, CA, USA). In brief, PCLS were incubated in 350µl TRIzol™ reagent (Thermo Fisher Scientific) for 30min on ice. After homogenization (Ultra-TURRAX), samples were centrifuged at 800g, 5min. Supernatant was mixed 1:1 (v/v) with 100% ethanol and loaded into a Zymo-Spin™ IIICG Column. The rest of the protocol was followed according to the manufacturer's instructions. cDNA was prepared from 100ng of RNA using iScript™ cDNA Synthesis Kit (Bio-Rad) in a Veriti™ 96-Well Thermal Cycler (Applied Biosystems). Quantitative RT-PCR was performed using SYBR green Advanced Master Mix and run on a ViiA 7 Real-Time PCR System (all from Applied Biosystems). Primers were designed in-house (see table S1) and synthesized by Eurofins Genomics. Data were normalized to the expression of *Hprt* using the following calculation: CT *Hprt* – CT gene of interest.

RNA sequencing

Snap frozen lung tissue was homogenized (T10 Ultra-Turrax Disperser) in TRIzol™ reagent (Thermo Fisher Scientific). Total RNA was extracted with chloroform, precipitated with isopropanol and washed in 80% ethanol. Subsequent library construction, sequencing and data analysis were performed by Novogene (Cambrigde, UK). In brief, RNA integrity and purity were checked using Agilent 5400. Messenger RNA was purified from total RNA using poly-T oligo-attached magnetic beads. After fragmentation, the first strand cDNA was synthesized using random hexamer primers followed by the second strand cDNA synthesis. The library was ready after end repair, A-tailing, adapter ligation, size selection, amplification, and purification. The library was checked with Qubit and real-time PCR for quantification and bioanalyzer for size distribution detection. Quantified libraries were pooled and sequenced on Illumina platforms, according to effective library concentration and data amount. Reference genome and gene model annotation files (Mus Musculus (GRCm38/mm10)) were downloaded from the genome website directly. Index of the reference genome was built and paired-end clean reads were aligned to the reference genome using Hisat2 v2.0.5. FeatureCounts v1.5.0-

p3 was used to count the reads numbers mapped to each gene. FPKM of each gene was calculated based on the length of the gene and reads count mapped to this gene.

Histology

Formalin-fixed paraffin embedded (FFPE) sections were prepared and dewaxed by xylene and decreasing concentration of ethanol before staining with hematoxylin and eosin or picrosirius red (PSR). PSR-stained sections were observed under polarized light on an Axio Scope.A1 (Zeiss) and pictures captured with a Gryphax camera (Jenoptik). Briefly, ten random fields were imaged (10x) with constant parameters among the samples. High resolution images were then analyzed using ImageJ (Bethesda, MD, USA).

Immunofluorescence staining and microscopy

FFPE lung tissue sections from PBS- and Bleomycin-treated mice, as well as from donors and IPF patients, were first placed in an incubator at 60°C for an hour, which was followed by tissue deparaffinization process. Using a Microm HMS 740 Robot-Stainer (Thermo Fisher Scientific), the slides containing the tissue sections were automatically incubated with Xylene (2x, 5min.), 100% EtOH (2x, 2min.), 90% EtOH (1x, 1min.), 80% EtOH (1x, 1min.), 70% EtOH (1x, 1min.), and distilled water (1x, 30sec.). Next, the tissue section containing slides were placed in R-Universal buffer (Aptum Biologics) followed by transfer to an antigen retrieval buffer containing pressure chamber (2100 Retrieval, Aptum Biologics). After 30 mins inside the pressure chamber, the slides were washed once in 1X Tris buffer for 10 min and then incubated in 5% BSA in PBS for 40 mins at room temperature. Subsequently, tissue sections were stained with primary antibodies overnight at 4°C under humid conditions. The following primary antibodies were used: anti-SFRP1 (Abcam, #ab126613, 1:100) and alpha-Smooth Muscle Actin (α-SMA) (Sigma-Aldrich, A5228, 1:500). Cell nuclei were stained with DAPI (40,6-diamidino-2-phenylindole, 1:2,000; Sigma-Aldrich). Next day, the slides were washed twice in 1X PBS for 10min, and further incubated with fluorescently-labeled secondary antibodies for 2 hours at room temperature under humid conditions. The following

fluorescently labeled secondary antibodies were used: donkey anti-rabbit IgG Alexa Fluor-568 (1:500; Invitrogen) and donkey anti-mouse IgG Alexa Fluor-488 (1:500; Invitrogen). Following two additional washes, slides were then counterstained with DAPI for 1 hour at room temperature, washed again two times with 1X PBS for 10min and subsequently dried at room temperature. Finally, tissue slides were mounted (Dako mounting medium) and kept in the dark at 4°C until further analysis. Images were acquired with an upright fluorescence microscope (AxioImager, Zeiss), equipped with an Axiocam and using the following objectives: Plan-Apochromat 20x/0.8 M27 and Plan-Apochromat 63x/1.4 M27.The automated microscopy system was driven by ZEN2009 (Zeiss) software. The final images were cropped and adjusted for contrast and brightness by using the ZEN2012 (Zeiss) software. The automated 3D-imaging of entire FFPE-mouse lung sections was accomplished using an Axioscan 7 (Zeiss) and a Plan-Apochromat 20×/0.8 M27 objective. The final images were analyzed, stitched, cropped and individually adjusted for contrast and brightness by using the ZEN2012 (Zeiss) software.

For AT2 experiments with rSFRP1, coverslips were immersed with PBS containing 0.1% Triton X-100 (Merck-Sigma Aldrich) for 15 minutes at RT, followed by three rinses in PBS for 5 minutes each. Subsequently, coverslips were treated with 300nM glycine (Merck-Sigma Aldrich) for 45mi at RT and then rinsed with PBS. Next, coverslips were incubated in blocking solution (PBS with 2% BSA, 0.1% Tween 20 and 0.1% Triton X-100) for 1 hour at RT to prevent unspecific binding of the antibodies and then rinsed with PBS. Primary antibodies, anti-Prosurfactant Protein C (proSP-C) antibody (Merck-Sigma Aldrich, diluted 1:200) and Krt8 (Developmental Studies Hybridoma Bank, #AB531826, diluted 1:200), were prepared in blocking solution and applied to coverslips, which were then incubated overnight at 4℃. On the subsequent day, coverslips were rinsed three times in PBS for 5 minutes each. Secondary antibodies, CF594 donkey anti-rabbit IgG (Biotium, #20152, diluted 1:500) and CF647 donkey anti rat IgG (Biotium, #20843, diluted 1:500), were diluted in blocking solution and applied to coverslips for an hour at RT. Following incubation, coverslips were rinsed three times in PBS

and mounted using mounting medium containing DAPI (Vector laboratories, #H-1800). Last, coverslips were imaged by an Olympus IX83 Microscope and the images were analyzed in QuPath (10).

Organoids were fixed with ice-cold methanol. Then, samples were blocked with 5% donkey normal serum in 0.1% PBST for 1h at RT, and incubated with primary antibodies diluted in 1% donkey normal serum at 4°C overnight. Organoids were washed 3X for 20 min with 0.1% PBST and then incubated for 2h at RT with secondary antibodies plus DAPI. Samples were washed again 3 times and imaged directly. Whole well images were acquired with an LSM 710 Confocal microscope (Zeiss, Germany) and mean fluorescence intensity was quantified using ImageJ (Fiji) (11).

Multiomic analyses

Mass spectrometry (MS) raw files were processed using the MaxQuant software (12). As previously described (13), peak lists were searched against the human UniProt FASTA database (November 2018), and a common contaminants database (247 entries) by the Andromeda search engine (14). Statistical and bioinformatics operations (such as normalization, imputation of missing values, annotation enrichment analysis, correlation analysis, hierarchical clustering, principal component analysis, and multiple-hypothesis testing corrections) were run with the Perseus software package (version 1.6.2.3.) (15). To determine compartment and cell type specific expression of EV specific proteins found by the MS analysis, the proteins were compared with a publicly available single cell RNA-sequencing dataset of whole lung from PBS and bleomycin-treated mice sampled with Dropseq (16). Data was analyzed using scanpy, a python package for the exploration of single-cell RNA-seq data (17). Different time points of bleomycin treatment were pooled into one category to compare PBS and bleomycin specific expression of POIs. To determine cell type specific expression of POIs in the Venn diagram, scanpy's tl.rank_genes_groups function was used to calculate significant cell type specific marker genes that were then matched with the EV specific POIs.

Scoring of EV protein gene signatures on compartments and cell types was done using scanpy's tl.score_genes function, which compares the average expression of signatures genes with randomly chosen reference genes. To determine the time component of Sfrp1 expression a recently published scRNAseq paper on mesenchyme enriched from bleomycin treated mouse lungs was used (16).

References

1. Satoh W, Gotoh T, Tsunematsu Y, Aizawa S, and Shimono A. Sfrp1 and Sfrp2 regulate anteroposterior axis elongation and somite segmentation during mouse embryogenesis. *Development.* 2006;133(6):989-99.
2. Uhl FE, Vierkotten S, Wagner DE, Burgstaller G, Costa R, Koch I, et al. Preclinical validation and imaging of Wnt-induced repair in human 3D lung tissue cultures. *Eur Respir J.* 2015;46(4):1150-66.
3. Mutze K, Vierkotten S, Milosevic J, Eickelberg O, and Konigshoff M. Enolase 1 (ENO1) and protein disulfide-isomerase associated 3 (PDIA3) regulate Wnt/beta-catenin-driven trans-differentiation of murine alveolar epithelial cells. *Dis Model Mech.* 2015;8(8):877-90.
4. Ng-Blichfeldt JP, de Jong T, Kortekaas RK, Wu X, Lindner M, Guryev V, et al. TGF-beta activation impairs fibroblast ability to support adult lung epithelial progenitor cell organoid formation. *Am J Physiol Lung Cell Mol Physiol.* 2019;317(1):L14-L28.
5. Mummler C, Burgy O, Hermann S, Mutze K, Gunther A, and Konigshoff M. Cell-specific expression of runt-related transcription factor 2 contributes to pulmonary fibrosis. *FASEB J.* 2018;32(2):703-16.
6. Oehrle B, Burgstaller G, Irmler M, Dehmel S, Grun J, Hwang T, et al. Validated prediction of pro-invasive growth factors using a transcriptome-wide invasion signature derived from a complex 3D invasion assay. *Sci Rep.* 2015;5:12673.
7. Hu Y, Ng-Blichfeldt JP, Ota C, Ciminieri C, Ren W, Hiemstra PS, et al. Wnt/beta-catenin signaling is critical for regenerative potential of distal lung epithelial progenitor cells in homeostasis and emphysema. *Stem Cells.* 2020;38(11):1467-78.
8. Lehmann M, Hu Q, Hu Y, Hafner K, Costa R, van den Berg A, et al. Chronic WNT/beta-catenin signaling induces cellular senescence in lung epithelial cells. *Cell Signal.* 2020;70:109588.
9. Burt M, Angelidou G, Mais CN, Preusser C, Glatter T, Heimerl T, et al. Lipid A in outer membrane vesicles shields bacteria from polymyxins. *J Extracell Vesicles.* 2024;13(5):e12447.
10. Bankhead P, Loughrey MB, Fernandez JA, Dombrowski Y, McArt DG, Dunne PD, et al. QuPath: Open source software for digital pathology image analysis. *Sci Rep.* 2017;7(1):16878.
11. Schindelin J, Arganda-Carreras I, Frise E, Kaynig V, Longair M, Pietzsch T, et al. Fiji: an open-source platform for biological-image analysis. *Nat Methods.* 2012;9(7):676-82.
12. Cox J, and Mann M. MaxQuant enables high peptide identification rates, individualized p.p.b.-range mass accuracies and proteome-wide protein quantification. *Nat Biotechnol.* 2008;26(12):1367-72.
13. Schiller HB, Fernandez IE, Burgstaller G, Schaab C, Scheltema RA, Schwarzmayr T, et al. Time- and compartment-resolved proteome profiling of the extracellular niche in lung injury and repair. *Mol Syst Biol.* 2015;11(7):819.
14. Cox J, Neuhauser N, Michalski A, Scheltema RA, Olsen JV, and Mann M. Andromeda: a peptide search engine integrated into the MaxQuant environment. *J Proteome Res.* 2011;10(4):1794-805.
15. Tyanova S, Temu T, Sinitcyn P, Carlson A, Hein MY, Geiger T, et al. The Perseus computational platform for comprehensive analysis of (prote)omics data. *Nat Methods.* 2016;13(9):731-40.
16. Mayr CH, Sengupta A, Ansari M, Pestoni JC, Ogar P, Angelidis I, et al. Autocrine Sfrp1 inhibits lung fibroblast invasion during transition to injury induced myofibroblasts. *BioRxiv.* 2022.
17. Wolf FA, Angerer P, and Theis FJ. SCANPY: large-scale single-cell gene expression data analysis. *Genome Biol.* 2018;19(1):15.

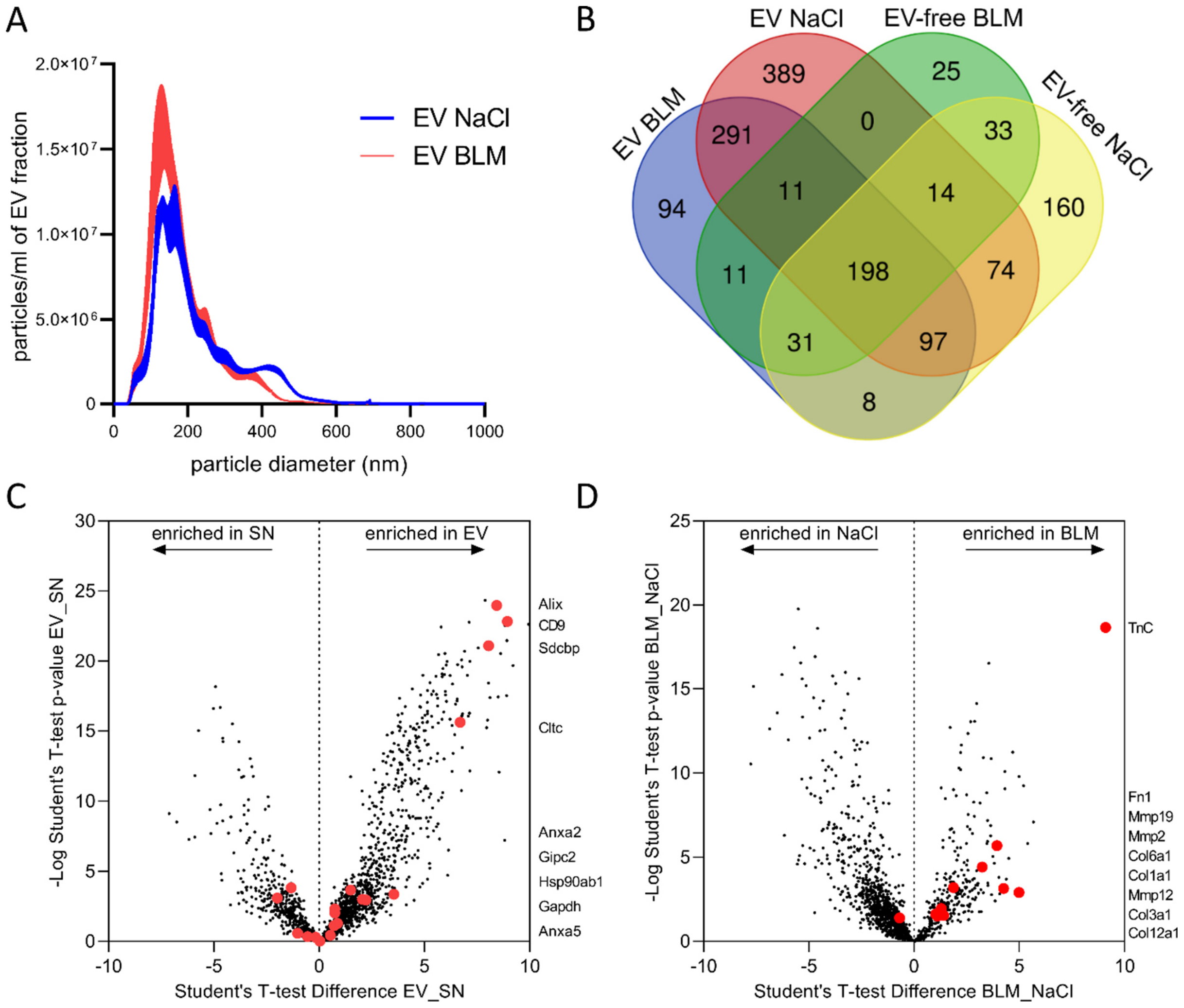

<u>Figure S1. Specific set of proteins are enriched in fibrotic EVs.</u> A) Nanoparticle tracking analysis of the EV samples used as input for the proteomic. **B)** Venn diagram showing the number of proteins identified in each fraction or overlapping over several fractions. **C, D)** Volcano plot depicting the proteins differentially regulated in NaCl vs BLM samples (C) or in EV vs EV-free fractions (D). Proteins classically enriched in EVs (Top20 based on Vesiclepedia), or fibrosis relevant proteins are highlighted in red in C and D, respectively.

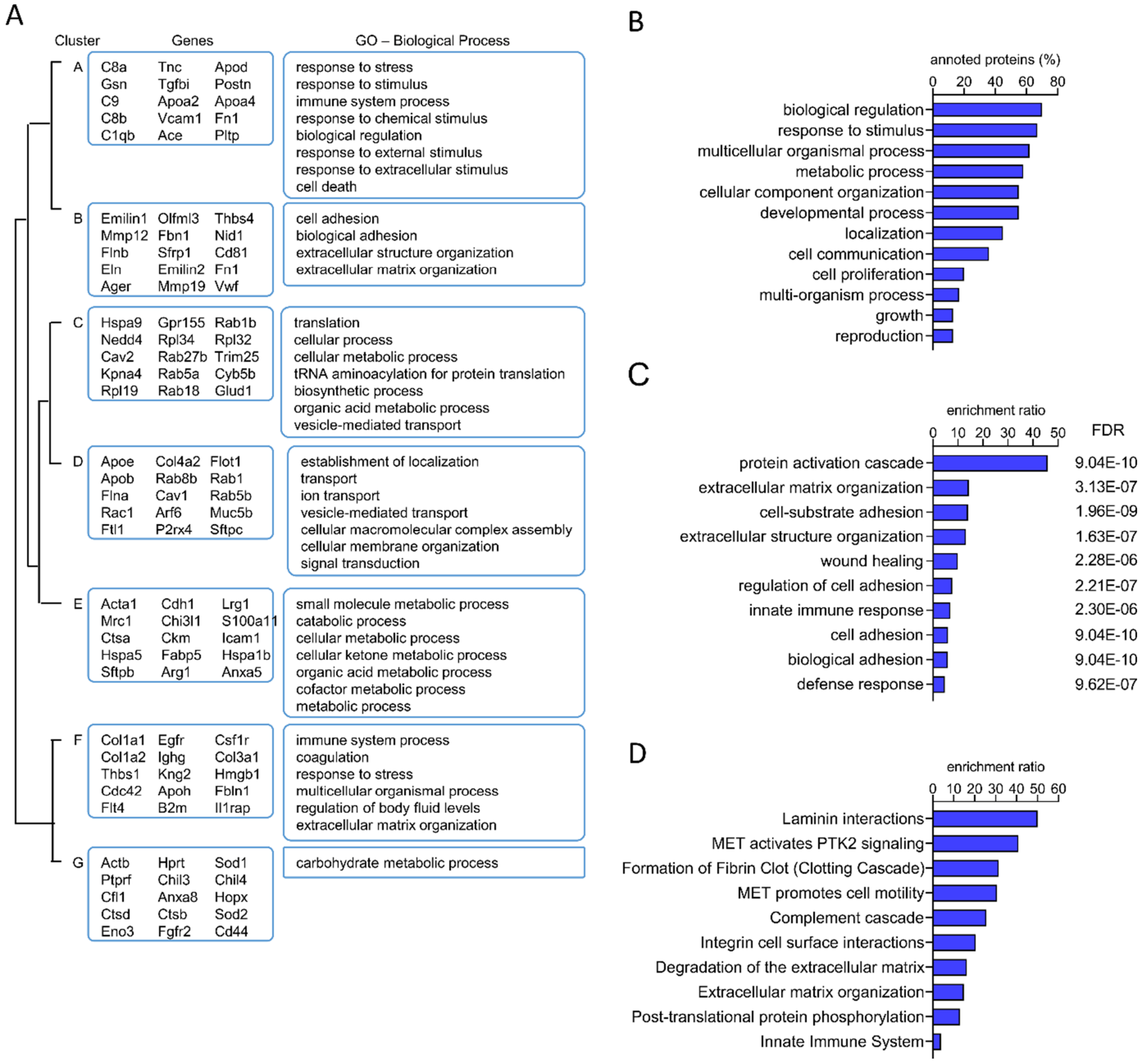

<u>Figure S2. GO annotations of the protein clusters.</u> A) GO analysis of the clusters identified in Figure 2. Top genes are highlighted for each cluster, as well as their GO term for biological process and molecular function. **B-D)** Gene ontology analysis of the 107 POIs for biological process (E), molecular process (F) or reactome study (G).

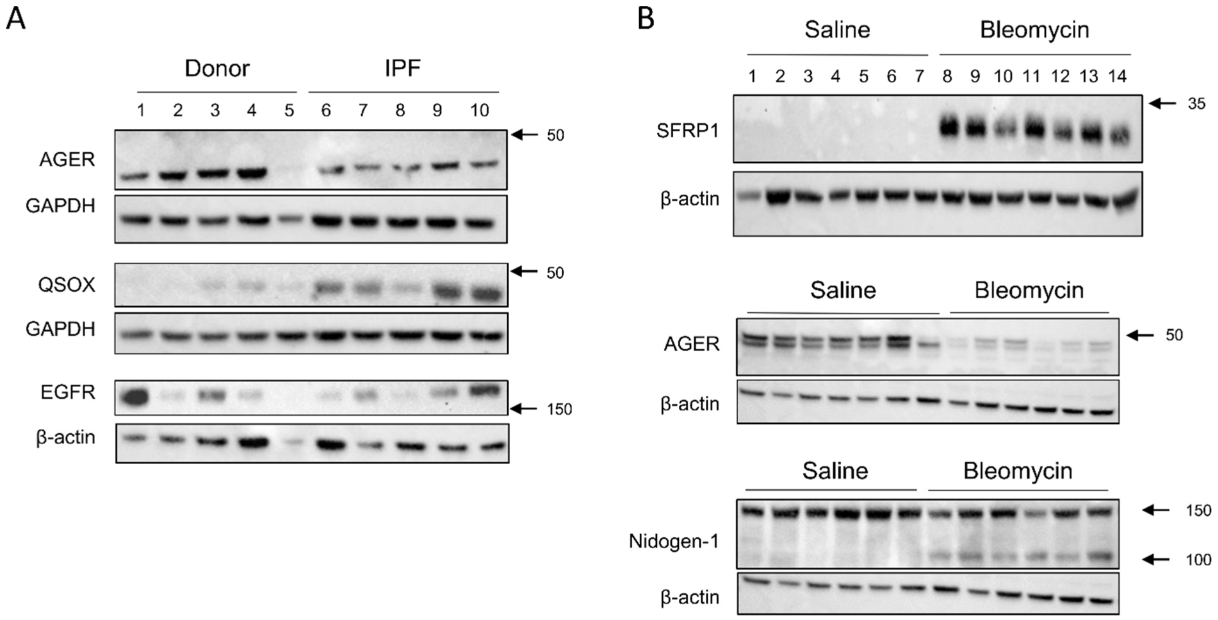

Figure S3. Proteins enriched in fibrotic EVs are dysregulated during pulmonary fibrosis. Expression of the top targets identified by the EV proteomic on tissue from **A)** patients with IPF or healthy donors or **B)** mice with bleomycin-induced pulmonary fibrosis and control mice. GAPDH and β-actin serve as loading control.

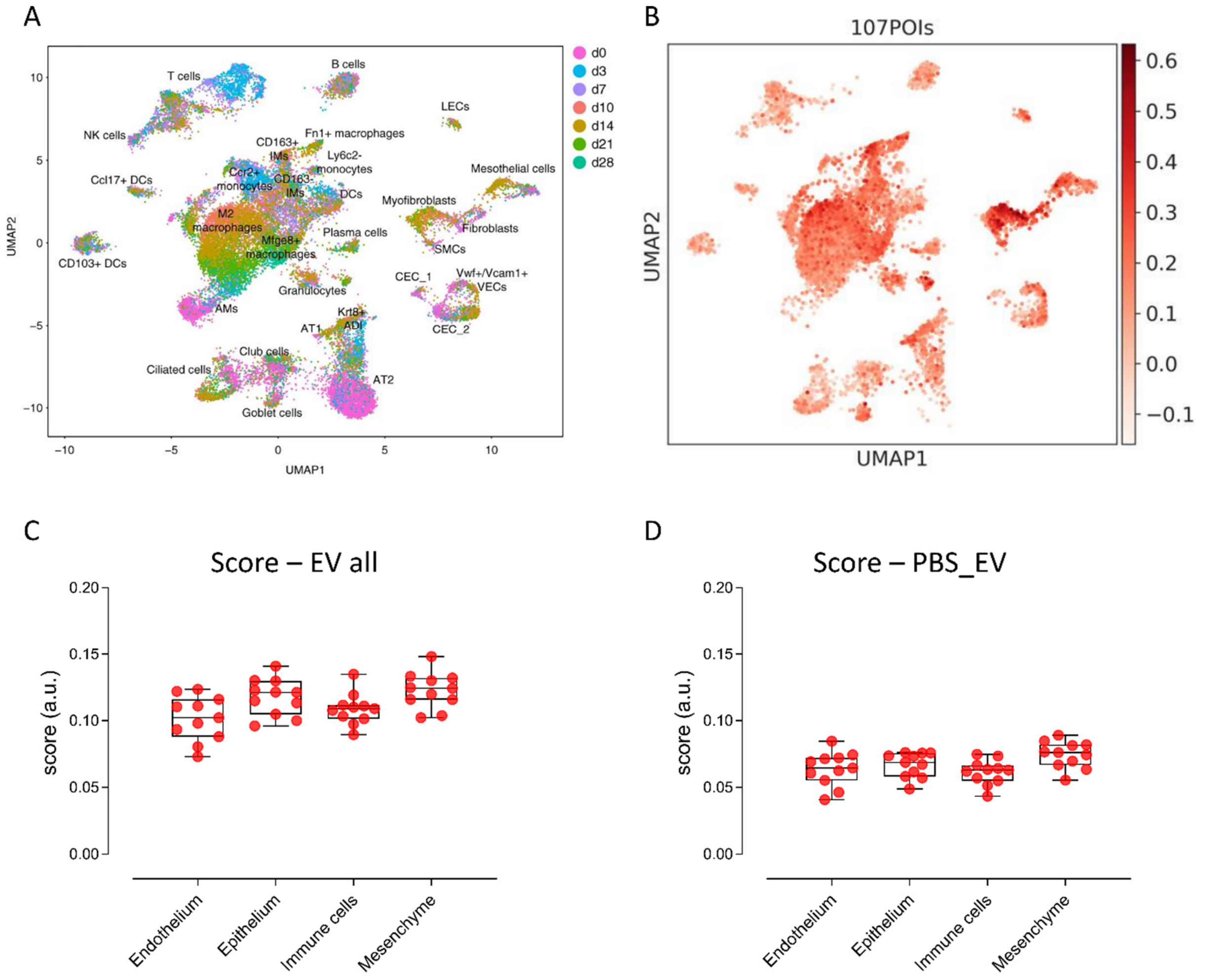

<u>Figure S4. Proteins linked to fibrotic EVs are expressed by mesenchymal cells.</u> A) Cell cluster from the scRNAseq dataset analyzed in Figure 3 and 4. Data extracted from GSE141259 (Strunz *et al.*, 2020). **B)** UMAP representation of the scRNAseq dataset with color coding indicating the scored mean expression of the 107 proteins specific to bleomycin BALF-EVs. **C-D)** Scoring of the scRNAseq dataset for the expression of the proteins identified in the EV samples (C) or specifically in PBS-BALF-EVs (D).

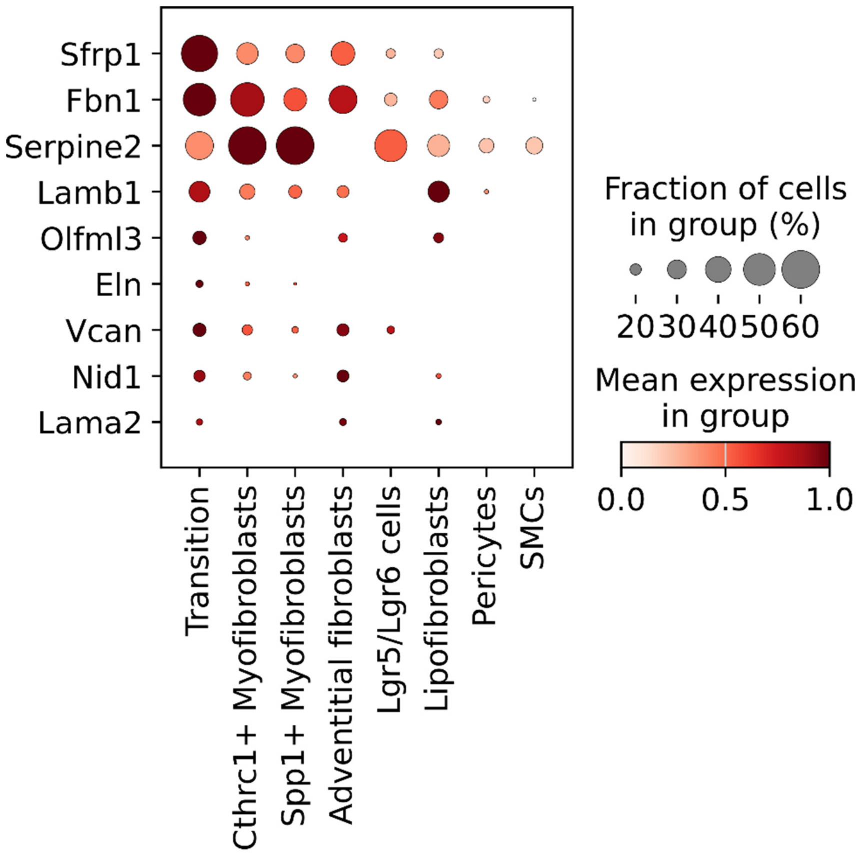

<u>Figure S5. The fibrotic EV protein SFRP1 is mainly expressed by pathological transitional fibroblasts.</u> Top proteins expressed in fibroblasts, among the proteins identified in bleomycin-BALF-EV, and classified among fibroblast subpopulations and other mesenchymal cell subsets. SMCs: smooth muscle cells.

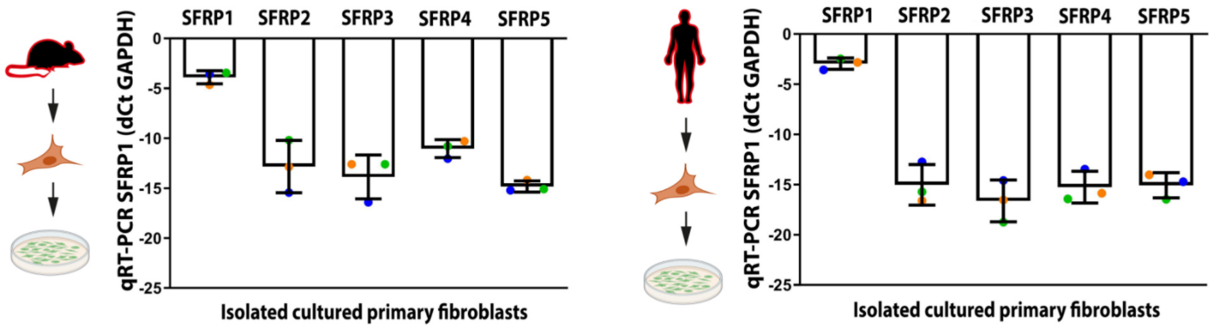

<u>Figure S6. SFRP1 is the SFRP isoform the most expressed in fibroblasts.</u> Primary mouse or human lung fibroblasts have been isolated from healthy animals/donors and cultured *in vitro*. The gene expression of SFRP members was assessed by qPCR. Data representative of three independent experiments.

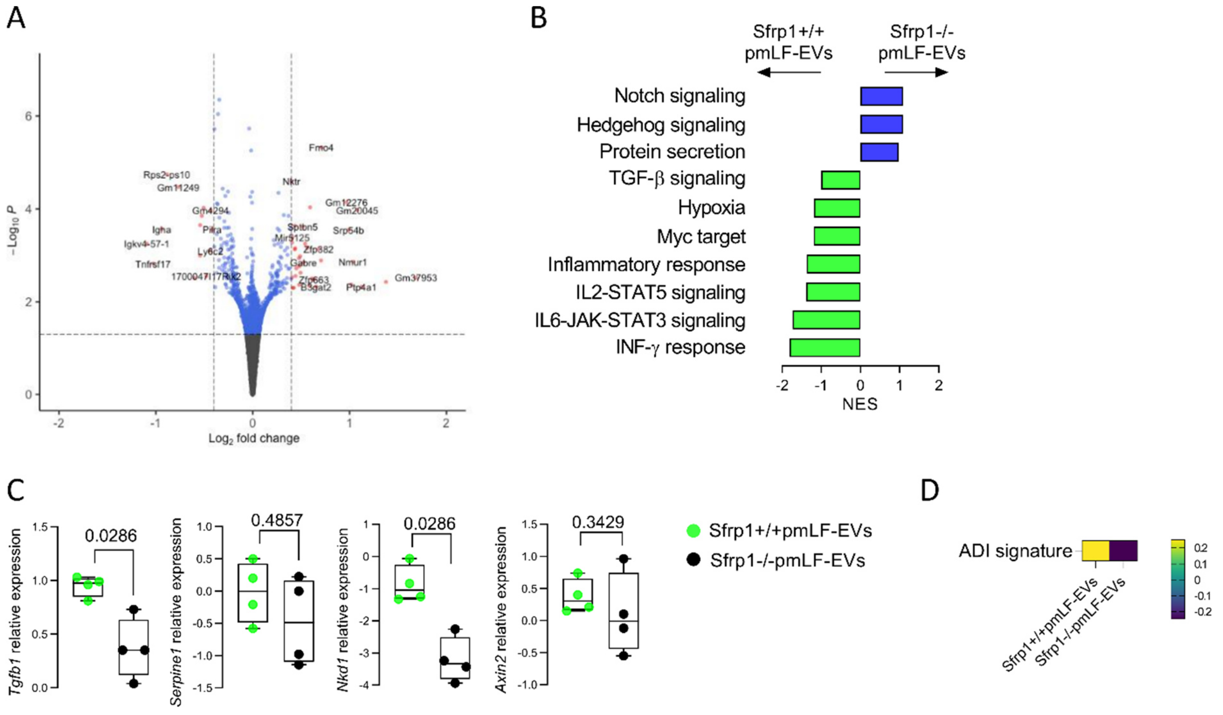

<u>Figure S7. Fibrosis-related mechanisms are increased in WT-EVs animals.</u> A) Volcano plot of the transcriptomic dataset between WT-EVs and KO-EVs. Number of significantly upregulated genes in each or the other group is shown. **B)** GSEA analysis on the differentially expressed genes in WT- (green) or KO-EVs (blue). Normalized enrichment scores for selected terms are shown. **C)** qPCR confirmation for the expression of *Tgfb1, Serpine1, Nkd1* and *Axin2* in WT-EVs (green) and KO-EVs (black). n=4 per group, each point represents a mouse sample. **D)** Heatmap for the ADI gene signature is shown. Statistical analysis by non-parametric Mann-Whitney. P values are indicated for each comparison.

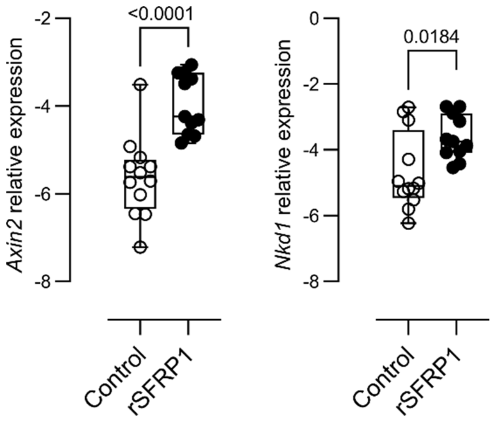

<u>Figure S8. SFRP1 promotes WNT/β-catenin signaling in AT2 cells.</u> Primary murine AT2 cells were cultured in presence of absence (control) of recombinant SFRP1 for 72h. RT-qPCR analysis for the expression of *Axin2* and *Nkd1*. Relative expressions normalized to Hprt are shown. Statistical analysis by non-parametric Mann-Whitney. P values are indicated for each comparison.

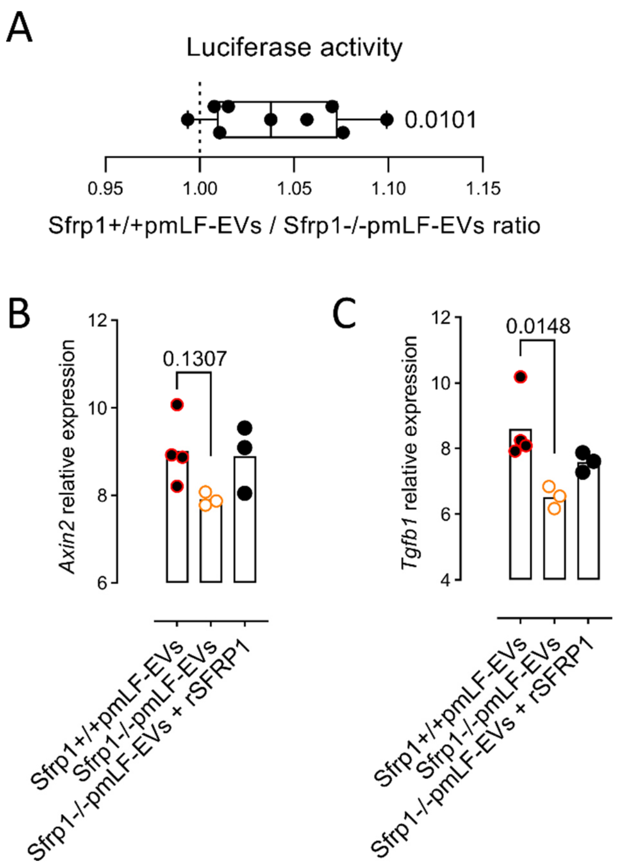

Figure S9. SFRP1 KO-EVs inhibits WNT/β-catenin and TGF-β signaling. A) WNT/β-catenin luciferase reporter 3T3 cells were cultured with WT- or (Sfrp1) KO-EVs. Luciferase signal was normalized to the signal measured in the KO-EV group. Each point represents a well of cells exposed to EVs. **B-C)** Organoids were analyzed by qPCR for the expression of *Axin2* (B) and *Tgfb1* (C). Each point is an organoid well. Statistical analysis by one sample t test (A) or one-way ANOVA with Kruskal-Wallis post-hoc test. P values are indicated for each comparison.

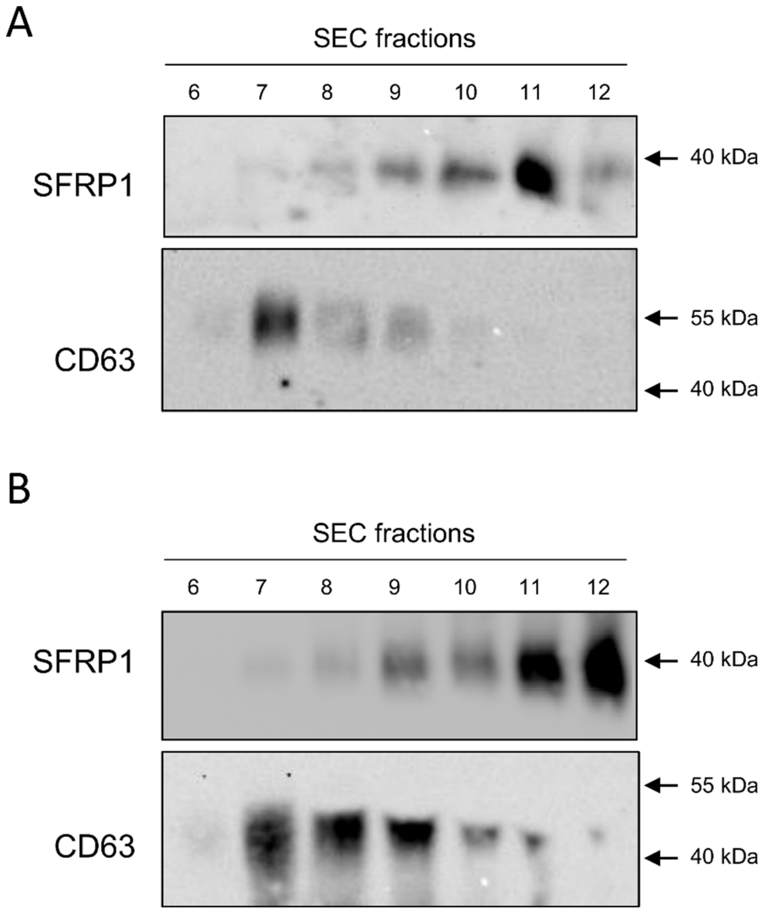

Figure S10. SFRP1 is secreted both in CD63 positive EVs and free. EVs were concentrated from culture media of phLF, from donor (A) and IPF (B) using SEC. Western Blot from fractions 6-12 shows protein expression of SFRP1 and CD63 using equal volumes per fraction. Molecular weight indicated.

Table S1. Primers used in this study.

	**Forward primer (5'->3')**	**Reverse primer (5'->3')**
Acta2	GAGACTCTCTTCCAGCCATCT	AGCATAGAGATCCTTCCTGATGT
Fn1	GGTGTAGCACAACTTCCAATTACG	GGAATTTCCGCCTCGAGTCT
Col1a1	AACGAGATCGAGCTCAGAGG	GACTGTCTTGCCCCAAGTTC
Col4a1	CTTCATTAGCAGGTGTGCGG	ACAGTGAGGACCAACCGTTA
Tgfb1	CGTGGCTTCTAGTGCTGACGC	CCATGTCGATGGTCTTGCAGGT
Serpine1	AGGTCAGGATCGAGGTAAACGAG	GGATCGGTCTATAACCATCTCCGT
Axin2	ACTGACCGACGATTCCATGT	TTTCTTACTCCCCATGCGGT
Nkd1	GACTGTGAGGAGGAGGCG	CAGCAAAGCTGTCACCTTCC
Krt8	CAAGGTGGAACTAGAGTCCCG	CTCGTACTGGGCACGAACTTC
Sprr1a	GCCCTGCACTGTACCTCCTC	GTGGCAGGGATCCTTGGTTTT
Itgb6	CAACTATCGGCCAACTCATTGA	GCAGTTCTTCATAAGCGGAGAT
Hprt	CCTAAGATGAGCGCAAGTTGAA	CCACAGGACTAGAACACCTGCTAA
