# Supplementary figures and images for: Fibroblast-derived extracellular vesicles contain SFRP1 and mediate pulmonary fibrosis
Source: JCI Insight. 2024 Aug 15;9(18):e168889. doi: 10.1172/jci.insight.168889 (PMC11457858; doi:10.1172/jci.insight.168889)

Figure 2D

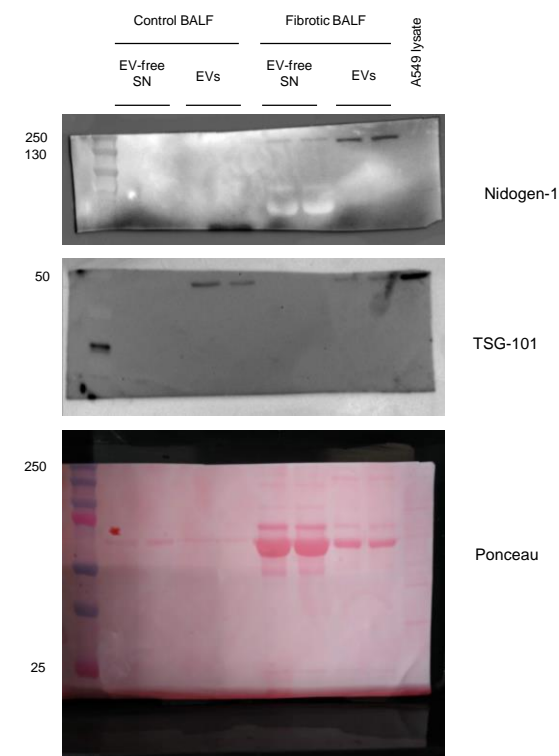

Figure 3I

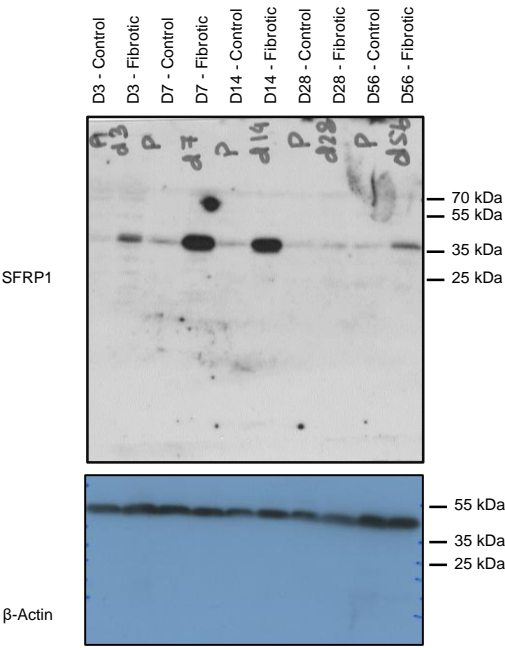

Figure 3K

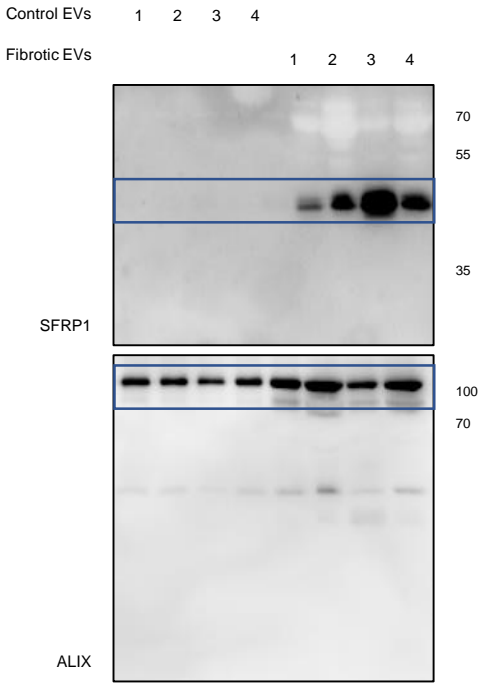

Figure 4A

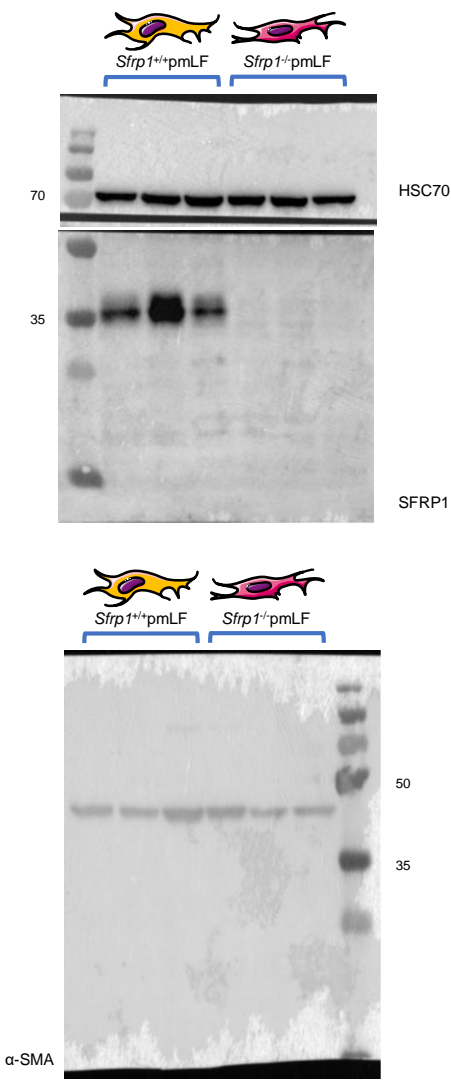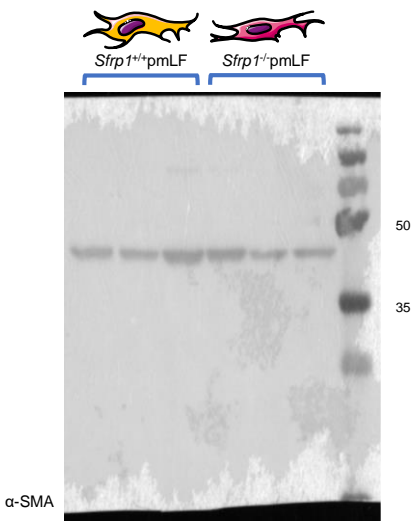

Figure 6C

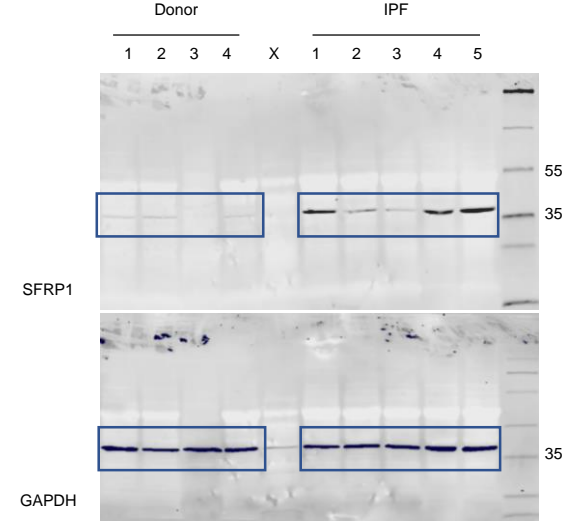

Figure 6D

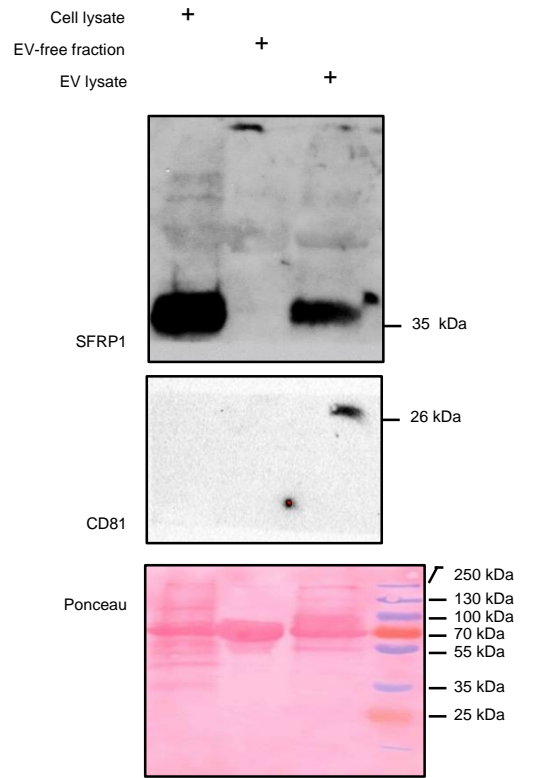

Figure S3A

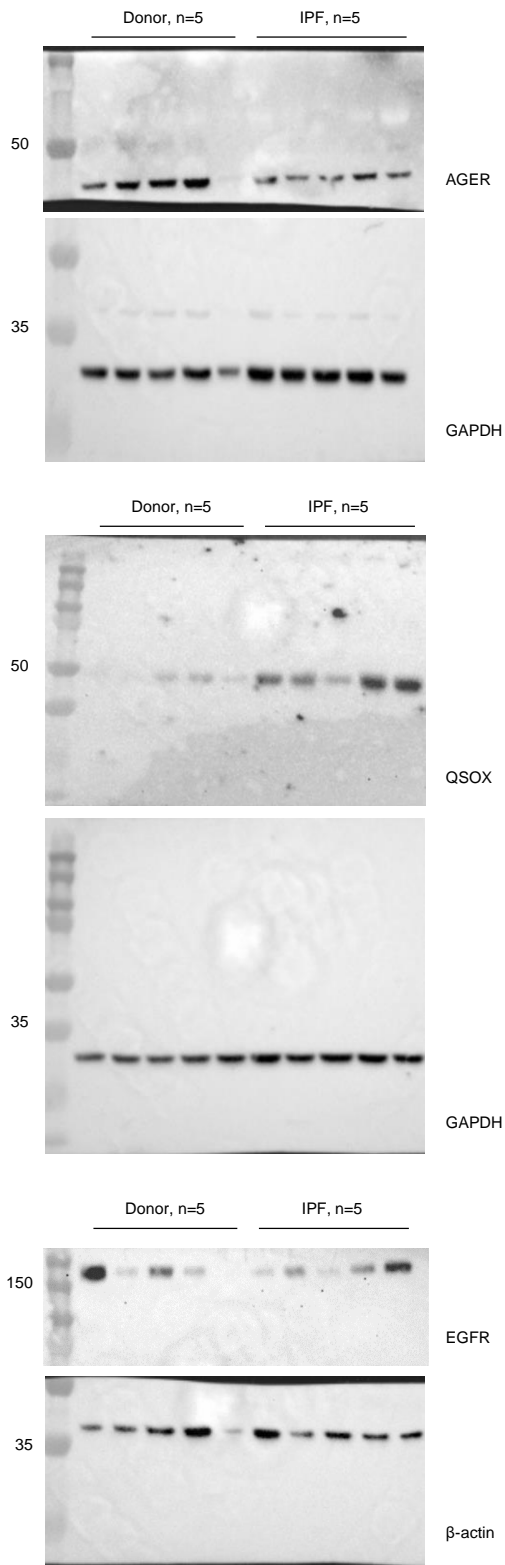

Figure S3B

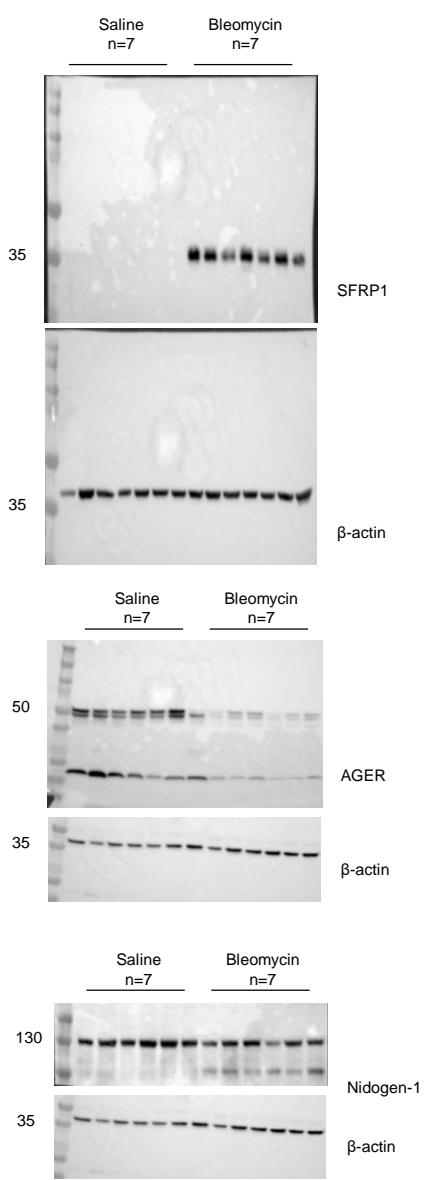

Figure S10A

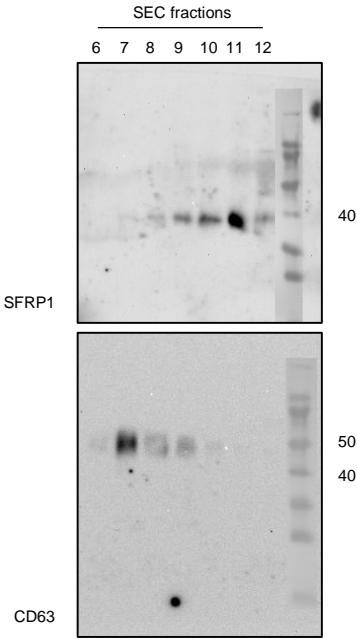

Figure S10B

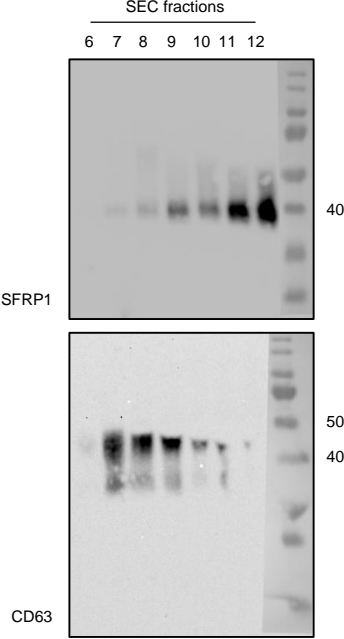

Supplement: Unedited blot and gel images [file jciinsight-9-168889-s188.pdf]
